# Supplementary figures and images for: Gene expression profiling identifies candidate biomarkers for new latent tuberculosis infections. A cohort study
Source: PLoS One. 2022 Sep 28;17(9):e0274257. doi: 10.1371/journal.pone.0274257 (PMC9518923; doi:10.1371/journal.pone.0274257)

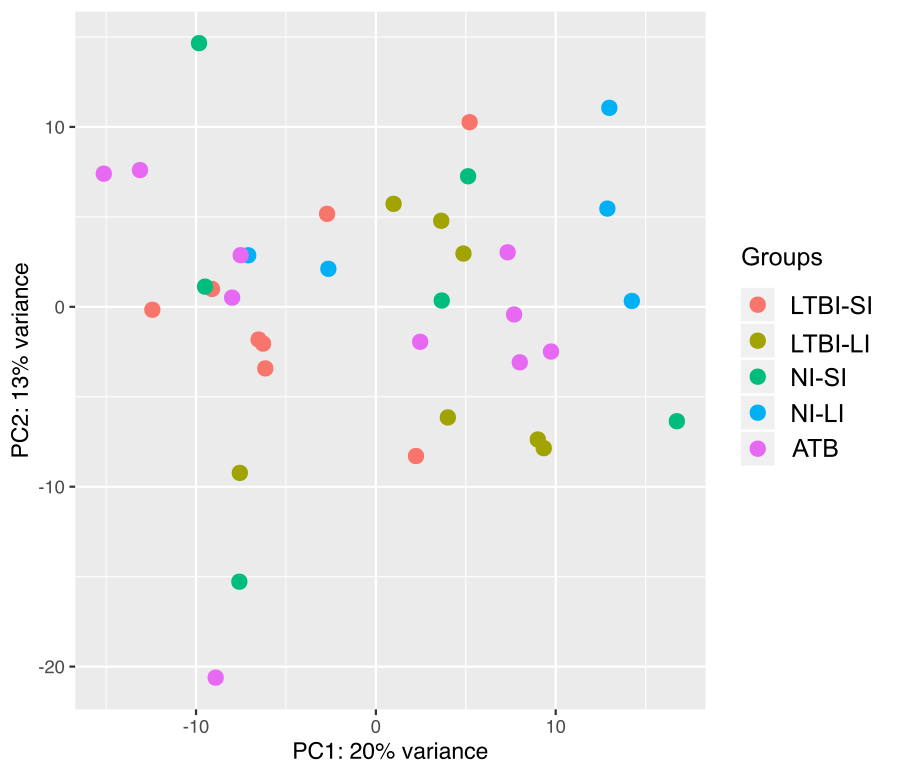

Supplement: S1 Fig — LTBI-LI: Latent tuberculosis infection with long incarceration (people already had ≥ 1 year in prison when entering the study). LTBI-SI: Latent tuberculosis infection with short incarceration (people started the follow-up with less than three months of incarceration). ATB: active tuberculosis. NI-SI: non-infected with short incarceration. NI-LI: non-infected with long incarceration. (TIF) [file pone.0274257.s001.tif]
