# Supplementary material for: Gene expression profiling identifies candidate biomarkers for new latent tuberculosis infections. A cohort study
Source: PLoS One. 2022 Sep 28;17(9):e0274257. doi: 10.1371/journal.pone.0274257 (PMC9518923; doi:10.1371/journal.pone.0274257)
Supplement: S1 Table — LTBI-LI: Latent tuberculosis infection with long incarceration (people already had ≥ 1 year in prison when entering the study). LTBI-SI: Latent tuberculosis infection with short incarceration (people started the follow-up with less than three months of incarceration). ATB: active tuberculosis. NI-SI: non-infected with short incarceration. NI-LI: non-infected with long incarceration. (PDF) [file pone.0274257.s002.pdf]

Differentially Expressed Genes

Contrast	Total	Up	Down
LTBI-SI_vs_NI-LI	235	119	116
LTBI-SI_vs_NI-SI	1	1	0
LTBI-SI_vs_ATB	18	1	17
LTBI-SI_vs_LTBI-LI	175	61	114
LTBI-LI_vs_NI-SI	5	1	4
LTBI-LI_vs_NI-LI	49	47	2
LTBI-LI_vs_ATB	23	13	10
NI-SI_vs_NI-LI	69	50	19
NI-SI_vs_ATB	0	0	0
NI-LI_vs_ATB	25	5	20
**Total ***	**600**	**298**	**302**

* Some genes could be in different categories

UP-REGULATED GENES			
**ensembl_gene_id**	**log2FoldChange**	**pvalue**	**padj**
ENSG00000278212.2	3.938696002	1.40961E-06	0.035592751

MAFF intera

DOWN-REGULATED GENES			
**ensembl_gene_id**	**log2FoldChange**	**pvalue**	**padj**

No genes found

acting protein

UP-REGULATED GENES			
ensembl_gene_id	log2FoldChange	pvalue	padj

No genes found

DOWN-REGULATED GENES			
ensembl_gene_id	log2FoldChange	pvalue	padj

No genes found

UP-REGULATED GENES			
**ensembl_gene_id**	**log2FoldChange**	**pvalue**	**padj**
ENSG00000176845.13	2.791395003	2.72802E-07	0.006888245

DOWN-REGULATED GENES			
**ensembl_gene_id**	**log2FoldChange**	**pvalue**	**padj**
ENSG00000173917.10	-1.067958514	7.9077E-06	0.099834661

LTBI-SI_vs_NI-LI

**UP-REGULATED GENES**			
**ensembl_gene_id**	**log2FoldChange**	**pvalue**	**padj**
ENSG00000049323	2.129370873	0.004544243	0.065050616
ENSG00000061656	1.117364251	0.007674069	0.082342303
ENSG00000076706	1.361455752	0.011602843	0.098768917
ENSG00000081041	2.607238764	0.001649412	0.041308744
ENSG00000085840	1.003290349	0.003180717	0.056185079
ENSG00000088899	1.044723358	0.008192504	0.085022219
ENSG00000090104	1.797810379	1.62268E-05	0.005468884
ENSG00000095794	1.597484117	0.004437752	0.06420871
ENSG00000100003	1.78869059	1.47967E-05	0.005468884
ENSG00000100024	1.855141625	0.000100328	0.012822469
ENSG00000100906	1.258352826	0.000328595	0.020634536
ENSG00000101665	1.224439774	0.006864287	0.077590868
ENSG00000102678	1.111294829	0.000545514	0.026299607
ENSG00000104660	1.067849461	1.15163E-07	0.000777292
ENSG00000104671	1.1856255	3.55339E-05	0.007965372
ENSG00000104856	1.22164021	0.003351429	0.057122399
ENSG00000104951	1.484029598	0.005583716	0.071445096
ENSG00000108342	7.777687905	4.91876E-06	0.004476539
ENSG00000108821	1.915150856	0.009077192	0.089570519
ENSG00000109929	1.173912168	0.000175315	0.015467792
ENSG00000112096	1.114381863	0.010819957	0.096116648
ENSG00000113448	1.359918819	0.001925142	0.044518787
ENSG00000115008	4.674068959	4.43578E-06	0.004476539
ENSG00000115009	3.930297772	4.14294E-05	0.008371331
ENSG00000116717	1.217345847	0.005926441	0.073328169
ENSG00000118503	1.299194679	0.00037017	0.021631704
ENSG00000118985	1.18712504	0.009697419	0.092058404
ENSG00000120875	1.925754492	0.004050052	0.061957899
ENSG00000121797	1.67305542	0.000986737	0.034066403
ENSG00000123342	2.338686183	0.001182065	0.036999427
ENSG00000123685	1.007045415	0.011679914	0.099243163
ENSG00000124145	1.85374398	0.011762976	0.099621253
ENSG00000124466	1.566445077	0.000508231	0.02540967
ENSG00000125084	1.236509058	0.00385692	0.060894219
ENSG00000125319	1.441238078	0.001788355	0.043032085
ENSG00000125538	3.316786388	6.05357E-05	0.01061262
ENSG00000125657	1.181548247	0.001573111	0.040818579
ENSG00000125726	1.199740849	0.000427404	0.023742926
ENSG00000127081	1.174168739	0.000389179	0.022355443
ENSG00000130340	1.009380434	0.000933997	0.033005309
ENSG00000130844	1.493423654	0.003243537	0.05666662
ENSG00000132819	1.039973386	0.001114596	0.035853969
ENSG00000134107	1.114895312	0.001607464	0.040864691
ENSG00000135047	1.668993354	0.006862335	0.077590868
ENSG00000136244	2.849217273	0.000287231	0.019233466
ENSG00000136603	1.276552462	3.82499E-05	0.008195791
ENSG00000136689	1.976615774	0.00584439	0.072779914
ENSG00000137507	1.954754742	0.00232241	0.048529742
ENSG00000138641	1.065406285	0.001381335	0.038847182

ENSG00000139289	2.098636793	0.002548643	0.0515804
ENSG00000139572	2.228632342	0.002620186	0.052309941
ENSG00000143333	2.667904469	0.00021667	0.016906483
ENSG00000145911	1.60179809	0.000323332	0.020634536
ENSG00000148344	3.989561009	1.06281E-05	0.005468884
ENSG00000152409	1.239361663	0.000203355	0.016437652
ENSG00000153234	1.229876755	0.005660491	0.071742982
ENSG00000154099	3.479676947	1.98129E-05	0.005714129
ENSG00000155380	1.229694664	4.83955E-05	0.00871054
ENSG00000158747	1.528066991	1.15476E-05	0.005468884
ENSG00000158747	1.528066991	1.15476E-05	0.005468884
ENSG00000160789	1.100692193	0.001242769	0.037760948
ENSG00000162433	2.143981132	0.009921555	0.092974969
ENSG00000162924	1.190016306	0.003238397	0.05666662
ENSG00000163600	1.007470748	0.001315738	0.038530361
ENSG00000163661	2.009215544	0.006212534	0.074478683
ENSG00000163734	2.710195711	0.00308059	0.055225607
ENSG00000164236	1.088243404	0.011021436	0.096924012
ENSG00000165410	1.082168484	0.000652664	0.028250607
ENSG00000165474	3.083778784	0.005051338	0.068876784
ENSG00000165997	1.146160283	0.000250878	0.018211685
ENSG00000166165	1.174207466	0.005368486	0.070471392
ENSG00000166451	1.04640396	0.009659464	0.091890842
ENSG00000166592	2.302292417	0.003049842	0.054955713
ENSG00000166920	3.317180043	0.000471142	0.024843524
ENSG00000170312	1.27110883	0.009506024	0.091462452
ENSG00000170989	1.071627715	4.60037E-05	0.008656916
ENSG00000172602	1.22199648	0.007167829	0.079581289
ENSG00000173391	2.954496007	0.000109643	0.013089371
ENSG00000173451	1.001425186	0.010625806	0.09530748
ENSG00000176105	1.281267087	0.00050771	0.02540967
ENSG00000176490	1.264153218	0.001581926	0.040818579
ENSG00000176845	3.443786067	8.44519E-06	0.005468884
ENSG00000178803	2.34705053	0.005371884	0.070471392
ENSG00000180611	1.13684686	0.006085076	0.074013946
ENSG00000182687	1.603068114	0.007982294	0.083985177
ENSG00000183484	1.05053759	0.002082455	0.045655597
ENSG00000183508	1.02904801	0.000303347	0.019970646
ENSG00000184163	1.055588494	0.000626046	0.027891093
ENSG00000184545	1.723396728	0.002069358	0.04556977
ENSG00000184588	1.065146952	0.006672691	0.07685551
ENSG00000185338	1.232970096	0.000193004	0.016106749
ENSG00000189057	1.375420006	0.004074299	0.062005595
ENSG00000197122	1.403909862	0.001641491	0.041186775
ENSG00000197147	1.100547945	8.59126E-08	0.000777292
ENSG00000197632	2.182142042	0.011136081	0.09748765
ENSG00000197860	1.003656413	1.87653E-06	0.003971196
ENSG00000204186	1.015913981	0.002747083	0.052813434
ENSG00000211665	3.132013103	0.006519513	0.076305102
ENSG00000217801	1.766607016	0.002968161	0.054071796
ENSG00000218018	1.097070953	0.00228104	0.04826294
ENSG00000220749	1.010954641	0.000362809	0.021386747
ENSG00000222009	1.232828142	0.004655233	0.065940173
ENSG00000230002	1.599173824	9.43777E-05	0.012615238
ENSG00000234883	1.244055399	0.0008166	0.031051512

ENSG00000237973	1.226607683	0.003519454	0.058508759
ENSG00000238039	1.762487187	0.002739738	0.052813434
ENSG00000253320	2.475907315	2.23671E-05	0.005982499
ENSG00000260317	1.007862556	0.008546822	0.087008707
ENSG00000260708	1.047826823	0.003728083	0.059627242
ENSG00000266709	1.040998712	0.002897085	0.053847263
ENSG00000267365	2.882231536	0.003332455	0.056905313
ENSG00000270681	1.537492489	0.004790619	0.066737421
ENSG00000271781	1.07187479	0.004996658	0.068477039
ENSG00000273951	1.676799044	1.94989E-05	0.005714129
ENSG00000274286	1.928220441	0.011388133	0.098350661
ENSG00000276070	2.129121643	0.00143513	0.039381572
ENSG00000276070	2.129121643	0.00143513	0.039381572
ENSG00000277632	1.793283541	0.003711134	0.059567888
ENSG00000278356	1.281417811	0.003402805	0.057489951

DOWN-REGULATED GENES

ensembl_gene_id	log2FoldChange	pvalue	padj
ENSG00000010704	-1.03458241	0.001601569	0.040850458
ENSG00000026559	-1.598401153	0.00088332	0.032140007
ENSG00000036448	-3.772378227	0.00030476	0.019970646
ENSG00000040633	-1.093357076	0.000931107	0.033005309
ENSG00000068001	-1.031627217	0.001590561	0.040818579
ENSG00000070371	-1.089527048	0.0015282	0.040532712
ENSG00000091106	-1.312379436	0.001023799	0.034208569
ENSG00000091972	-1.145589892	0.010047535	0.093378157
ENSG00000092067	-1.518558835	0.006416804	0.075585026
ENSG00000103196	-1.202242355	0.011254656	0.097803498
ENSG00000106624	-1.074346302	0.005716578	0.07205237
ENSG00000107566	-1.062069037	9.79672E-06	0.005468884
ENSG00000109814	-1.109001257	0.000457606	0.024584576
ENSG00000112195	-1.114880136	0.000589654	0.027073931
ENSG00000114737	-1.643239868	0.001852062	0.043715893
ENSG00000117115	-1.604767085	0.005950782	0.073561907
ENSG00000119121	-1.635895303	0.009309011	0.090776477
ENSG00000119457	-1.420977395	0.00946151	0.091300212
ENSG00000120093	-1.099492147	6.6497E-05	0.011014979
ENSG00000121716	-1.482282423	0.000360574	0.021348172
ENSG00000121807	-2.013407713	0.000216472	0.016906483
ENSG00000121858	-1.367060361	0.000106847	0.012879039
ENSG00000121931	-1.225934621	0.000526385	0.025703392
ENSG00000123405	-1.262991517	0.003258654	0.05666662
ENSG00000123427	-1.382222693	8.95535E-05	0.012615238
ENSG00000125863	-1.268036182	0.000170729	0.015364479
ENSG00000131471	-1.222523519	0.002886943	0.053827139
ENSG00000132763	-1.236878735	0.009966308	0.09314553
ENSG00000133063	-1.664533463	0.007336903	0.080691858
ENSG00000133561	-1.148443839	0.000679716	0.028655159
ENSG00000135407	-1.165938815	8.49946E-05	0.012471112
ENSG00000136630	-1.349023787	0.002148041	0.046468589
ENSG00000139174	-1.370508157	0.000560469	0.02653597

ENSG00000139679	-1.200280076	0.000713882	0.029290854
ENSG00000139998	-1.242424762	0.005049678	0.068876784
ENSG00000142405	-1.350326153	0.001603878	0.040850458
ENSG00000143457	-1.013315062	0.000162004	0.014876793
ENSG00000143878	-1.008992282	0.003901061	0.061161923
ENSG00000145723	-1.027483495	0.00299588	0.054503214
ENSG00000147437	-1.195064268	1.30523E-05	0.005468884
ENSG00000147592	-1.060835409	0.011611695	0.098768917
ENSG00000149534	-1.8357915	0.008808801	0.088216543
ENSG00000152213	-1.223439899	0.002851378	0.05362246
ENSG00000154734	-1.836898776	0.000169355	0.015364479
ENSG00000157551	-1.28946777	0.009175638	0.090146968
ENSG00000158715	-1.453225528	0.00557399	0.071445096
ENSG00000160113	-1.154453323	0.000511978	0.025502571
ENSG00000160285	-1.104987441	2.94184E-06	0.003971196
ENSG00000160318	-1.021116114	0.008505323	0.086813589
ENSG00000162222	-1.181719723	0.000270534	0.018832616
ENSG00000162747	-1.876912827	0.005865435	0.072907462
ENSG00000163154	-1.082637383	0.011535458	0.098768917
ENSG00000163393	-1.072603801	4.07431E-05	0.008371331
ENSG00000163464	-1.802198729	0.003441238	0.057634333
ENSG00000163563	-1.346601493	0.002809574	0.053267474
ENSG00000163606	-1.016780739	0.008256533	0.085471577
ENSG00000165118	-1.300278358	0.003428291	0.057631739
ENSG00000165646	-1.066487576	0.009341476	0.0908282
ENSG00000166398	-1.006050981	0.005129059	0.069387888
ENSG00000167210	-1.32931005	0.009624927	0.091890282
ENSG00000168329	-1.593766568	0.001703651	0.042197393
ENSG00000170915	-1.690065658	4.4938E-05	0.008656916
ENSG00000171115	-1.183576889	0.000853921	0.031820163
ENSG00000173110	-1.871620386	0.000430114	0.023795507
ENSG00000173535	-1.620719179	0.006768973	0.077465828
ENSG00000173917	-1.417073668	1.49386E-05	0.005468884
ENSG00000174007	-1.267887611	0.001963874	0.044683481
ENSG00000174123	-1.243844325	0.002134237	0.046342481
ENSG00000174600	-1.130600243	0.006904362	0.077733092
ENSG00000175857	-1.007272569	0.004921332	0.067824614
ENSG00000176222	-1.366904097	0.007953496	0.083958851
ENSG00000178966	-1.301753004	9.50144E-05	0.012615238
ENSG00000179889	-1.11131272	0.000496379	0.025296041
ENSG00000179889	-1.11131272	0.000496379	0.025296041
ENSG00000180340	-1.610343889	0.004013438	0.061878069
ENSG00000180871	-2.011094696	0.000917781	0.032688996
ENSG00000180953	-1.001390902	0.001973907	0.044752147
ENSG00000181631	-1.447119287	0.002647509	0.052326096
ENSG00000182700	-1.114826165	0.005470837	0.070872371
ENSG00000183307	-1.432134943	0.007688794	0.082342303
ENSG00000183625	-1.607887403	0.006882238	0.07767837
ENSG00000183734	-1.104719606	0.00163399	0.041074909
ENSG00000186205	-1.433344881	0.004692968	0.066322201
ENSG00000187210	-1.384421526	0.000185996	0.015692234
ENSG00000188305	-1.716742251	0.001431535	0.039381572
ENSG00000197182	-1.244285081	0.001283231	0.038224839
ENSG00000197520	-1.36926295	0.000261699	0.018691433
ENSG00000198440	-1.185184956	0.000137928	0.013791802

ENSG00000198538	-1.116050892	0.007294318	0.080562237
ENSG00000198736	-1.064623025	0.004062826	0.061957899
ENSG00000211789	-1.229951275	0.008585196	0.087009403
ENSG00000215271	-1.263687462	0.000278461	0.018984593
ENSG00000225101	-1.290870607	0.002931901	0.053847263
ENSG00000225194	-1.076799919	0.011820399	0.099699897
ENSG00000235314	-1.08193017	0.000680768	0.028655159
ENSG00000238113	-1.130721557	0.001904908	0.044315713
ENSG00000242028	-1.082482767	0.002085232	0.045655597
ENSG00000244482	-1.333361238	0.011393992	0.098350661
ENSG00000250251	-1.398923369	0.000142058	0.013834945
ENSG00000250510	-1.478831341	0.001144768	0.03653244
ENSG00000254837	-2.018518619	1.26403E-05	0.005468884
ENSG00000254838	-1.006696627	0.002138485	0.046342481
ENSG00000254860	-1.056237863	0.001171217	0.036999427
ENSG00000260828	-1.183264088	0.000776049	0.030364887
ENSG00000261087	-1.130736762	0.001131291	0.03618791
ENSG00000263961	-1.347673281	0.00771165	0.082342303
ENSG00000272668	-1.51348757	0.001029186	0.034219176
ENSG00000272688	-1.059820622	1.72003E-05	0.005468884
ENSG00000272908	-1.255039746	0.001415497	0.039235721
ENSG00000274536	-1.041311546	0.00410322	0.062034238
ENSG00000275111	-1.817056353	0.000619219	0.027891093
ENSG00000276550	-1.005605336	0.004164541	0.062118385
ENSG00000278195	-1.145914888	0.00363215	0.059072762
ENSG00000280670	-1.489835746	0.010722834	0.095716629
ENSG00000280832	-1.118420363	0.001213679	0.037441893
ENSG00000281162	-1.719802315	0.001907358	0.044315713

LTBI-SI_vs_NI-SI

UP-REGULATED GENES

ensembl_gene_id	log2FoldChange	pvalue	padj
ENSG00000211639	-4.429421181	5.0041E-06	0.061571809

OWN-REGULATED GENES

ensembl_gene_id	log2FoldChange	pvalue	padj

No genes found

LTBI-SI_vs_ATB

UP-REGULATED GENES

ensembl_gene_id	log2FoldChange	pvalue	padj
ENSG00000166165	1.281370855	0.000258274	0.097669706

DOWN-REGULATED GENES

ensembl_gene_id	log2FoldChange	pvalue	padj
ENSG00000005381	-2.338364614	0.000114042	0.076972551
ENSG00000036448	-3.310383998	0.000142444	0.082136233
ENSG00000079385	-1.300770338	0.000289229	0.097669706
ENSG00000086548	-3.480289502	2.65175E-05	0.050981802
ENSG00000115271	-1.147228442	0.000146031	0.082136233
ENSG00000118113	-2.927698844	0.000166028	0.089648503
ENSG00000121807	-2.012004018	9.44826E-06	0.050981802
ENSG00000121858	-1.189622438	5.2037E-05	0.070244751
ENSG00000133063	-2.038897485	9.15054E-05	0.076972551
ENSG00000134827	-2.247709292	0.000108826	0.076972551
ENSG00000149516	-2.297447801	1.9771E-05	0.050981802
ENSG00000157551	-1.492990961	0.000296648	0.097669706
ENSG00000163563	-1.477575427	8.18281E-05	0.073639821
ENSG00000178966	-1.169211097	4.14315E-05	0.062142588
ENSG00000185745	-1.563371838	7.4886E-05	0.073639821
ENSG00000254837	-1.445059138	0.00026399	0.097669706
ENSG00000272398	-1.587570269	0.000134824	0.082136233

LTBI-LI_vs_NI-SI

UP-REGULATED GENES

ensembl_gene_id	log2FoldChange	pvalue	padj
ENSG00000237604	3.430097721	1.05086E-05	0.095485995

DOWN-REGULATED GENES

ensembl_gene_id	log2FoldChange	pvalue	padj
ENSG00000104267	-2.524402087	2.02344E-05	0.095485995
ENSG00000176749	-1.069317357	1.83493E-05	0.095485995
ENSG00000205426	-2.08051658	8.00426E-06	0.095485995
ENSG00000220793	-2.985524023	2.27005E-05	0.095485995

LTBI-LI_vs_NI-LI

UP-REGULATED GENES

ensembl_gene_id	log2FoldChange	pvalue	padj
ENSG00000048462	3.198641653	2.79568E-06	0.005671049
ENSG00000071539	1.772459593	0.000219555	0.078433761
ENSG00000099958	2.031537633	7.33718E-05	0.043061385
ENSG00000110777	1.09619803	0.000240744	0.084173208
ENSG00000115884	3.71154531	5.54953E-06	0.007599614
ENSG00000132465	2.919849627	4.38703E-05	0.029585296
ENSG00000133328	3.096652887	2.50018E-06	0.005671049
ENSG00000135916	1.467809279	0.000153532	0.065779359
ENSG00000143603	2.454980217	0.000100945	0.051838633
ENSG00000157456	1.896591018	0.000203127	0.074177592
ENSG00000170312	1.863014587	0.000180946	0.069150648
ENSG00000170476	2.205573866	4.95455E-07	0.001463587
ENSG00000178445	3.268119632	1.65943E-05	0.016838655
ENSG00000180535	3.574607214	9.30606E-05	0.050326599
ENSG00000183010	1.823705043	0.000177864	0.069150648
ENSG00000211592	2.587198152	6.57727E-08	0.000540421
ENSG00000211597	1.633135063	2.0691E-05	0.017000778
ENSG00000211632	2.546486342	3.84103E-05	0.028690757
ENSG00000211640	3.029867238	0.000148705	0.065779359
ENSG00000211659	3.158470233	1.17498E-05	0.013344257
ENSG00000211662	2.981599211	6.33848E-05	0.038577855
ENSG00000211668	2.886405115	4.16754E-05	0.029585296
ENSG00000211677	2.604130849	1.92104E-05	0.017000778
ENSG00000211679	2.468205321	0.000261303	0.088307923
ENSG00000211896	2.931458928	6.77764E-09	0.000111377
ENSG00000211897	3.207818419	1.66379E-07	0.000911366
ENSG00000211935	4.752009091	2.68351E-05	0.020999094
ENSG00000211937	3.269489585	0.000147953	0.065779359
ENSG00000222037	1.992951963	0.000156112	0.065779359
ENSG00000224041	3.032055072	1.74197E-05	0.016838655
ENSG00000230006	2.17342902	1.98075E-05	0.017000778
ENSG00000237649	1.864098578	0.000185167	0.069155597
ENSG00000239571	3.458574168	9.49385E-05	0.050326599
ENSG00000239951	2.245093396	0.000263317	0.088307923
ENSG00000239975	3.454910524	3.10591E-06	0.005671049
ENSG00000242076	2.279637058	0.000176249	0.069150648
ENSG00000242371	2.303736445	4.5009E-05	0.029585296
ENSG00000242534	3.381686822	2.2796E-07	0.000936517
ENSG00000243238	2.517728811	0.000124096	0.059978362
ENSG00000243466	2.016439441	5.72992E-05	0.036215312
ENSG00000244116	2.595898647	1.21806E-05	0.013344257
ENSG00000244437	1.887341204	0.00027131	0.089168598
ENSG00000244575	2.675458367	5.25323E-06	0.007599614
ENSG00000251546	3.432131927	4.88492E-06	0.007599614
ENSG00000254709	1.887014303	5.34383E-07	0.001463587
ENSG00000258572	1.877872446	0.000135923	0.063817659
ENSG00000278196	2.033374352	0.000307336	0.099028575

DOWN-REGULATED GENES			
**ensembl_gene_id**	**log2FoldChange**	**pvalue**	**padj**
ENSG00000120093	-1.117680833	8.51016E-05	0.048223284
ENSG00000173917	-1.476480199	1.16473E-05	0.013344257

LTBI-LI_vs_ATB

UP-REGULATED GENES

ensembl_gene_id	log2FoldChange	pvalue	padj
ENSG00000060140	1.377906225	0.000453067	0.077557862
ENSG00000130812	1.37557428	0.000655921	0.091758343
ENSG00000131400	1.122533121	0.00029257	0.063438875
ENSG00000170476	1.465462243	7.07668E-05	0.036899981
ENSG00000211892	2.112778798	0.000134892	0.046182853
ENSG00000211896	2.189457038	2.65163E-07	0.003449774
ENSG00000211897	1.973870255	0.000127791	0.046182853
ENSG00000211898	1.193868996	0.000441168	0.076527939
ENSG00000222037	1.812837039	3.58876E-05	0.027464559
ENSG00000230006	1.606007712	0.000163138	0.051766577
ENSG00000239571	3.130656729	2.11272E-05	0.027464559
ENSG00000243466	1.645108256	9.24639E-05	0.03784874
ENSG00000254709	1.440878471	4.68234E-06	0.014858532

DOWN-REGULATED GENES

ensembl_gene_id	log2FoldChange	pvalue	padj
ENSG00000038945	-1.737219767	0.00077695	0.096267749
ENSG00000047634	-1.001109845	1.3173E-05	0.021422661
ENSG00000067646	-1.030757784	8.73386E-06	0.018937925
ENSG00000116016	-2.008334393	0.000259137	0.061297604
ENSG00000134970	-1.007693483	3.54225E-05	0.027464559
ENSG00000144290	-1.642949321	4.28728E-05	0.02827273
ENSG00000176749	-1.107081692	6.08207E-07	0.003956384
ENSG00000196126	-5.065059349	0.000286359	0.063438875
ENSG00000211821	-2.088017877	0.000287812	0.063438875
ENSG00000247627	-1.257971879	8.47723E-05	0.037526602

NI-SI_vs_NI-LI

UP-REGULATED GENES			
ensembl_gene_id	log2FoldChange	pvalue	padj
ENSG00000048462	2.466358766	0.00047566	0.085634454
ENSG00000049323	2.692857763	0.000660855	0.093238282
ENSG00000071539	1.886435187	0.00012237	0.055858664
ENSG00000073282	1.888249509	0.000796768	0.098972487
ENSG00000089685	1.919362891	0.00040549	0.085634454
ENSG00000094804	1.85763814	0.000535247	0.089679753
ENSG00000100024	2.470678106	8.45574E-07	0.011004851
ENSG00000104267	2.48134106	0.000118983	0.055858664
ENSG00000108342	6.978766416	8.87808E-05	0.055858664
ENSG00000109805	1.497121413	0.000705833	0.095755465
ENSG00000111206	1.791943066	0.000229952	0.076462496
ENSG00000115008	4.064848124	0.000163367	0.065478118
ENSG00000117724	1.29610144	0.000672433	0.093644819
ENSG00000135476	2.006711846	7.82983E-05	0.055858664
ENSG00000143603	2.256888258	0.0004741	0.085634454
ENSG00000145386	2.352943641	8.00941E-05	0.055858664
ENSG00000146670	1.746633828	0.000144989	0.059565265
ENSG00000150681	2.547455897	0.000265263	0.077707217
ENSG00000157456	2.029563144	0.000110914	0.055858664
ENSG00000158406	1.710911953	0.00046877	0.085634454
ENSG00000161888	1.260223418	0.000767131	0.097722995
ENSG00000162433	3.064760839	0.000439416	0.085634454
ENSG00000167513	1.574240369	0.000317068	0.079425823
ENSG00000170312	2.245639419	1.00658E-05	0.027568472
ENSG00000170476	1.667589502	0.000236815	0.076462496
ENSG00000171848	1.785890058	0.00066384	0.093238282
ENSG00000175746	5.214804231	0.000118033	0.055858664
ENSG00000187699	2.687451224	0.000317538	0.079425823
ENSG00000197147	1.021953783	2.62119E-06	0.011004851
ENSG00000198888	1.036602317	0.000823829	0.099543969
ENSG00000211592	1.728585535	0.000482463	0.085634454
ENSG00000211638	5.053799267	3.57474E-06	0.011748744
ENSG00000211639	4.753944985	1.35079E-05	0.031710744
ENSG00000211648	2.65142692	0.000436201	0.085634454
ENSG00000211662	3.134132124	4.76513E-05	0.049010002
ENSG00000211669	2.803218047	0.000269538	0.077707217
ENSG00000211673	1.961396137	0.000513928	0.088898794
ENSG00000211676	2.06112794	0.000644004	0.092502805
ENSG00000211677	2.325716581	0.000223404	0.076462496
ENSG00000211896	1.819008257	0.000505523	0.088375068
ENSG00000211897	2.708002619	1.93356E-05	0.039717782
ENSG00000227165	2.512856207	0.000647345	0.092502805
ENSG00000229344	3.15747105	0.000114213	0.055858664
ENSG00000233968	2.18933639	0.000726084	0.096223756
ENSG00000234618	1.002882386	0.000119403	0.055858664
ENSG00000235065	1.415802207	0.000765197	0.097722995
ENSG00000238201	4.157849996	5.03522E-05	0.049010002
ENSG00000253320	2.433671711	8.5672E-05	0.055858664
ENSG00000277075	2.096728086	0.000562383	0.089679753
ENSG00000280079	1.687964874	0.000107471	0.055858664

DOWN-REGULATED GENES			
**ensembl_gene_id**	**log2FoldChange**	**pvalue**	**padj**
ENSG00000107317	-1.885880962	0.000258982	0.077379195
ENSG00000109956	-1.524845687	0.000318999	0.079425823
ENSG00000142173	-1.114118341	0.000558931	0.089679753
ENSG00000147234	-1.386785327	5.0701E-05	0.049010002
ENSG00000156886	-2.863575774	0.000352741	0.084467377
ENSG00000160318	-1.447516669	0.000455912	0.085634454
ENSG00000169926	-1.595387201	0.000132542	0.057019701
ENSG00000173930	-1.219011243	0.000409024	0.085634454
ENSG00000176083	-2.141211994	0.000302961	0.079425823
ENSG00000178162	-3.736178777	1.83738E-06	0.011004851
ENSG00000178537	-1.043497861	0.000395171	0.085634454
ENSG00000181036	-1.288854276	0.000831176	0.0996986
ENSG00000183542	-2.152159908	0.00059376	0.089679753
ENSG00000183734	-1.521106976	4.68006E-05	0.049010002
ENSG00000197057	-1.155180777	0.000440169	0.085634454
ENSG00000204525	-5.266149922	0.000251338	0.076485836
ENSG00000237604	-3.232460534	8.44331E-05	0.055858664
ENSG00000255441	-1.547854477	0.000393466	0.085634454
ENSG00000278420	-1.81403345	0.000620392	0.090220426

NI-SI_vs_ATB

UP-REGULATED GENES			
ensembl_gene_id	log2FoldChange	pvalue	padj

No genes found

DOWN-REGULATED GENES			
ensembl_gene_id	log2FoldChange	pvalue	padj

No genes found

NI-LI_vs_ATB

UP-REGULATED GENES

ensembl_gene_id	log2FoldChange	pvalue	padj
ENSG00000142173	1.046406148	0.000338988	0.083159606
ENSG00000173917	1.183561733	0.000161535	0.064013238
ENSG00000178162	2.293724959	0.000269639	0.074853881
ENSG00000269403	1.838102471	7.7956E-05	0.04302894
ENSG00000287200	1.351666668	2.2243E-05	0.031251381

DOWN-REGULATED GENES

ensembl_gene_id	log2FoldChange	pvalue	padj
ENSG00000067646	-1.028771018	6.46507E-05	0.037828743
ENSG00000073282	-1.891315301	0.000303177	0.078093352
ENSG00000090104	-1.598763435	6.60871E-05	0.037828743
ENSG00000102554	-1.416267709	3.86545E-05	0.034532358
ENSG00000108342	-6.742731387	4.70373E-05	0.03634806
ENSG00000115008	-3.620267776	0.000226232	0.068557251
ENSG00000121742	-1.962737413	0.000348599	0.084181329
ENSG00000125538	-2.861689007	0.000316278	0.080132392
ENSG00000134970	-1.289697556	2.10868E-06	0.006517934
ENSG00000136603	-1.120141208	0.000172048	0.064853681
ENSG00000141655	-1.136886234	0.000463537	0.096810285
ENSG00000154165	-2.254270976	0.000374739	0.086369566
ENSG00000156113	-2.178344528	0.000503347	0.098612184
ENSG00000165997	-1.04690544	0.000504067	0.098612184
ENSG00000173391	-2.793380471	0.000144338	0.062214681
ENSG00000185338	-1.407341602	9.50913E-06	0.018370442
ENSG00000213386	-3.52227117	0.000271227	0.074853881
ENSG00000217801	-2.151444559	0.000168576	0.064853681
ENSG00000247627	-1.315500602	0.000222991	0.068557251
ENSG00000286330	-1.060188485	0.000211333	0.066887439

LTBI-SI_vs_LTBI-LI

UP-REGULATED GENES			
**ensembl_gene_id**	**log2FoldChange**	**pvalue**	**padj**
ENSG00000061656	1.125240346	0.003105921	0.065597459
ENSG00000090104	1.365230839	0.000308478	0.029509514
ENSG00000095794	1.725034503	0.000714561	0.036029616
ENSG00000100003	1.487087533	6.8328E-05	0.01779034
ENSG00000100024	1.10936062	0.00866843	0.098871528
ENSG00000101665	1.448826503	0.000427424	0.031821029
ENSG00000104951	1.398100166	0.00397653	0.072364938
ENSG00000108821	1.951317412	0.003399631	0.068785696
ENSG00000109929	1.212705621	1.96663E-05	0.010939392
ENSG00000112137	1.208048294	0.008265982	0.097452777
ENSG00000113448	1.442473143	0.000290556	0.028209914
ENSG00000114315	1.303089051	0.002062152	0.055660714
ENSG00000116285	1.246285533	0.001977612	0.054741178
ENSG00000117519	1.007183534	0.007495203	0.093652678
ENSG00000119508	2.305565231	0.00542079	0.081245133
ENSG00000119986	1.317115653	0.008076011	0.096914798
ENSG00000121797	1.322175646	0.004018473	0.072364938
ENSG00000124466	1.169261725	0.004164132	0.073408336
ENSG00000125084	1.300247892	0.000822949	0.038574168
ENSG00000130844	1.335548984	0.003731003	0.070965414
ENSG00000135114	1.014500296	0.004872781	0.078014891
ENSG00000137507	2.147359696	0.000232726	0.025101278
ENSG00000143333	1.897004223	0.003556571	0.069915783
ENSG00000144115	1.636713069	0.004657921	0.076615175
ENSG00000144290	1.654291952	7.85738E-05	0.018399952
ENSG00000145911	1.754088506	1.46231E-05	0.01056924
ENSG00000148344	2.143502139	0.007797817	0.095202761
ENSG00000152409	1.123843644	0.000205573	0.024320696
ENSG00000153234	1.174167212	0.003617472	0.070454055
ENSG00000158747	1.635630617	2.38392E-07	0.001857494
ENSG00000158747	1.635630617	2.38392E-07	0.001857494
ENSG00000162924	1.238524045	0.00073915	0.036452087
ENSG00000166920	2.622217359	0.002271683	0.057714998
ENSG00000167034	1.008363433	0.007897179	0.095683918
ENSG00000167618	1.298028781	0.008610869	0.098871528
ENSG00000168994	1.141598824	0.004522889	0.076018343
ENSG00000170889	1.13190914	0.002791433	0.06367827
ENSG00000171617	1.046049721	0.004963182	0.078122461
ENSG00000172602	1.116649378	0.006665345	0.08921414
ENSG00000173391	2.03330696	0.003073068	0.065115013
ENSG00000176105	1.154675158	0.000545181	0.03421266
ENSG00000176490	1.015597789	0.004971107	0.078122461
ENSG00000177374	1.193594933	0.00108153	0.041629322
ENSG00000182853	1.050432567	0.007898888	0.095683918
ENSG00000183484	1.105047108	0.000362158	0.031568186
ENSG00000183813	1.020855949	0.00163823	0.050813356
ENSG00000184545	1.620097188	0.001409341	0.048124745
ENSG00000188290	1.1612618	0.007935959	0.095959553
ENSG00000196126	4.394824137	0.002724908	0.063244605
ENSG00000198369	1.389885215	0.005918166	0.083807859
ENSG00000204001	4.042462481	0.000570807	0.03421266
ENSG0000218018	1.147444415	0.000445012	0.031821029

ENSG00000234883	1.294567246	0.000126587	0.021869029
ENSG00000237973	1.086696371	0.004396364	0.075261215
ENSG00000237989	1.011117343	0.004618465	0.076497705
ENSG00000250321	1.029398799	0.004926275	0.078122461
ENSG00000260317	1.087759078	0.001821819	0.052900071
ENSG00000266709	1.088765493	0.000607789	0.034530523
ENSG00000274677	1.235122309	0.005889238	0.083807859
ENSG00000275302	1.241935706	0.008119606	0.09697025
ENSG00000283199	1.023776893	0.000692659	0.035894092

DOWN-REGULATED GENES

ensembl_gene_id	log2FoldChange	pvalue	padj
ENSG00000003249	-1.495995728	0.000351416	0.031530527
ENSG00000007038	-1.433682835	0.005107593	0.078638806
ENSG00000008516	-1.487707601	0.007272655	0.092374154
ENSG00000026559	-1.227420215	0.005180588	0.079480477
ENSG00000048462	-2.227516911	0.000199781	0.024320696
ENSG00000079385	-1.053841081	0.007106299	0.091521097
ENSG00000087586	-1.1226843	0.001593224	0.050555726
ENSG00000089685	-1.282246732	0.005349532	0.080782509
ENSG00000091106	-1.211769453	0.000892845	0.039925398
ENSG00000091972	-1.243680046	0.002128776	0.056406052
ENSG00000092067	-1.416553348	0.005462305	0.081309593
ENSG00000092758	-1.106460388	0.004778749	0.077424068
ENSG00000099958	-1.344492626	0.002816293	0.06367827
ENSG00000100311	-1.327957471	0.000979683	0.040807577
ENSG00000100721	-1.458077509	0.000578538	0.03421266
ENSG00000111261	-1.15456496	0.008799391	0.099288882
ENSG00000111291	-2.803767532	0.00033336	0.031307368
ENSG00000116729	-1.185299687	0.005357136	0.080782509
ENSG00000116985	-1.016741924	0.008572082	0.098871528
ENSG00000117399	-1.329602316	0.004884138	0.078014891
ENSG00000120049	-1.053647634	0.0014871	0.049104504
ENSG00000121807	-1.94222111	8.72393E-05	0.01842104
ENSG00000121858	-1.142040273	0.000368879	0.031568186
ENSG00000123405	-1.266056364	0.001167454	0.043598244
ENSG00000125863	-1.00397665	0.001097163	0.041859502
ENSG00000130812	-1.455208826	0.000611582	0.034530523
ENSG00000131400	-1.187215555	0.000281309	0.027937616
ENSG00000132465	-2.222240725	0.000432804	0.031821029
ENSG00000133063	-2.149988138	0.000140219	0.023091705
ENSG00000133328	-2.571268819	6.30903E-06	0.006840035
ENSG00000134057	-1.160174133	0.001480721	0.049018266
ENSG00000134061	-1.36511244	0.001829145	0.052900071
ENSG00000135898	-1.032811483	0.001740918	0.051593032
ENSG00000135916	-1.214411942	0.000392748	0.031736965
ENSG00000136630	-1.142911306	0.004252669	0.074364543
ENSG00000139174	-1.119734445	0.002229934	0.057334852
ENSG00000142405	-1.206924623	0.00193961	0.0546828
ENSG00000142583	-1.073224246	0.000152837	0.023935787
ENSG00000142675	-1.026888058	8.0711E-06	0.007500356

ENSG00000143457	-1.037766173	2.27028E-05	0.010939392
ENSG00000150967	-1.122445334	0.002125601	0.056406052
ENSG00000152213	-1.332598405	0.000356087	0.031568186
ENSG00000157551	-1.288296875	0.004198562	0.073697825
ENSG00000159339	-1.101545111	0.001666894	0.051195624
ENSG00000162747	-1.84713886	0.002824179	0.06367827
ENSG00000163154	-1.038267173	0.007694862	0.094532724
ENSG00000163563	-1.441416307	0.000427826	0.031821029
ENSG00000164124	-1.28447323	0.003444714	0.069074384
ENSG00000164403	-1.364438965	0.005630709	0.082681185
ENSG00000166123	-1.013377102	8.77867E-05	0.01842104
ENSG00000166851	-1.220080503	0.000286614	0.028036491
ENSG00000168268	-1.162575072	0.003531717	0.069915783
ENSG00000169116	-1.146625707	0.000702206	0.035947315
ENSG00000170476	-1.832100985	2.26041E-06	0.004201137
ENSG00000170909	-1.115467045	0.002919211	0.06432282
ENSG00000170915	-1.311198303	0.000509494	0.033458165
ENSG00000173110	-1.766577505	0.000257022	0.026632142
ENSG00000173535	-1.491536217	0.006117599	0.084580189
ENSG00000174007	-1.24532471	0.000896019	0.039925398
ENSG00000174123	-1.199830874	0.001136219	0.042971541
ENSG00000175857	-1.069795737	0.001015346	0.041103468
ENSG00000178445	-2.562330655	0.000104632	0.019728473
ENSG00000178966	-1.181554304	0.000127751	0.021869029
ENSG00000180549	-1.042731155	0.00542967	0.081245133
ENSG00000180871	-1.611884353	0.003454945	0.069074384
ENSG00000181631	-1.341057274	0.002178221	0.056931599
ENSG00000183010	-1.562374011	0.000233455	0.025101278
ENSG00000183307	-1.428764234	0.003567739	0.069915783
ENSG00000185432	-1.011791844	4.51643E-05	0.015770427
ENSG00000186205	-1.2713027	0.005851831	0.083807859
ENSG00000186529	-1.42807125	0.002271052	0.057714998
ENSG00000187210	-1.349578641	6.3051E-05	0.017089437
ENSG00000187554	-1.114036695	0.000982258	0.040807577
ENSG00000188305	-1.422293311	0.004033013	0.07243869
ENSG00000211592	-1.854976491	1.17364E-05	0.009543137
ENSG00000211640	-2.652732289	0.000165479	0.024189622
ENSG00000211659	-1.991822959	0.001698673	0.051323935
ENSG00000211662	-2.453364301	0.000192026	0.024320696
ENSG00000211668	-2.319160209	0.000190574	0.024320696
ENSG00000211669	-1.998917795	0.002285567	0.057741229
ENSG00000211677	-1.689198651	0.001708168	0.051323935
ENSG00000211896	-2.252332465	4.63044E-07	0.001857494
ENSG00000211897	-2.310380945	1.96781E-05	0.010939392
ENSG00000211898	-1.01462876	0.004456027	0.075736043
ENSG00000211934	-2.409626589	0.000889785	0.039925398
ENSG00000211937	-2.361668301	0.00169367	0.051323935
ENSG00000211941	-1.748057989	0.006461745	0.087376672
ENSG00000222037	-1.74583265	0.000152551	0.023935787
ENSG00000228427	-1.025266386	0.00081326	0.038474578
ENSG00000230006	-1.761108323	8.45737E-05	0.018399952
ENSG00000239571	-2.859228489	0.000211408	0.024320696
ENSG00000239855	-1.621378455	0.004327224	0.074664703
ENSG00000239951	-1.836220649	0.000729878	0.036382064
ENSG00000240382	-2.07627665	0.001829749	0.052900071

ENSG00000240864	-1.407666323	0.005790542	0.083752676
ENSG00000241351	-1.471204087	0.00152511	0.049708942
ENSG00000241755	-1.749900267	0.006295927	0.086028638
ENSG00000242371	-1.906719972	0.000121852	0.021422826
ENSG00000242534	-2.231323632	6.25653E-05	0.017089437
ENSG00000243238	-1.831538688	0.001517105	0.049591792
ENSG00000243466	-2.098129269	2.14474E-06	0.004201137
ENSG00000244116	-1.502696558	0.003456374	0.069074384
ENSG00000244437	-1.550653771	0.000697355	0.035894092
ENSG00000244575	-2.087634354	4.93494E-05	0.016050895
ENSG00000251546	-2.197537292	0.000760723	0.036929139
ENSG00000253239	-2.041385243	0.008443738	0.098371729
ENSG00000254709	-1.656554163	5.71097E-07	0.001857494
ENSG00000254837	-1.678009604	8.48576E-05	0.018399952
ENSG00000257275	-1.226555619	0.007597305	0.094348413
ENSG00000260528	-1.109987864	0.008575539	0.098871528
ENSG00000269981	-1.177553059	0.001085831	0.041671551
ENSG00000275111	-1.507544716	0.001962174	0.0546828
ENSG00000278196	-1.506604456	0.002391011	0.05877357
ENSG00000281162	-1.374743631	0.006947696	0.091102557
