# Supplementary material for: Gene expression profiling identifies candidate biomarkers for new latent tuberculosis infections. A cohort study
Source: PLoS One. 2022 Sep 28;17(9):e0274257. doi: 10.1371/journal.pone.0274257 (PMC9518923; doi:10.1371/journal.pone.0274257)
Supplement: S2 Table — LTBI-LI: Latent tuberculosis infection with long incarceration (people already had ≥ 1 year in prison when entering the study). LTBI-SI: Latent tuberculosis infection with short incarceration (people started the follow-up with less than three months of incarceration). ATB: active tuberculosis. NI-SI: non-infected with short incarceration. NI-LI: non-infected with long incarceration. (PDF) [file pone.0274257.s003.pdf]

ATB group

ID - DOWN GENES	Symbol	Description	Literature review
1 ENSG00000060140.9	STYK1	serine/threonine/tyrosine kinase 1	Not Reported
1 ENSG00000211892.4	IGHG4	immunoglobulin heavy constant gamma 4	Not Reported
1 ENSG00000269403.1	AC008750.8	novel transcript	Not Reported
1 ENSG00000287200.1	AC022506.2	novel transcript, antisense to INHBEand GLI1	Not Reported

ID - UP GENES	Symbol	Description	Literature review
1 ENSG00000005381.8	MPO	myeloperoxidase	Kaforou, 2013 - up in TB compared with LTBI
1 ENSG00000038945.15	MSR1	macrophage scavenger receptor 1	Not Reported
1 ENSG00000047634.15	SCML1	Scm polycomb group protein like 1	Not Reported
1 ENSG00000067646.12	ZFY	zinc finger protein Y-linked	Not Reported
1 ENSG00000086548.9	CEACAM6	CEA cell adhesion molecule 6	Not Reported
1 ENSG00000102554.14	KLF5	Kruppel like factor 5	Not Reported
1 ENSG00000115271.11	GCA	grancalcin	Not Reported
1 ENSG00000116016.14	EPAS1	endothelial PAS domain protein 1	Not Reported
1 ENSG00000118113.12	MMP8	matrix metallopeptidase 8	Not Reported
1 ENSG00000121742.19	GJB6	gap junction protein beta 6	Not Reported
1 ENSG00000134827.8	TCN1	transcobalamin 1	Not Reported
1 ENSG00000134970.14	TMED7	transmembrane p24 trafficking protein 7	Walter ND, 2016 - up, it distinguishes activeTB from pneumonia or latent TB infection
1 ENSG00000141655.17	TNFRSF11A	TNF receptor superfamily member 11a	Not Reported
1 ENSG00000149516.14	MS4A3	membrane spanning 4-domains A3	Not Reported
1 ENSG00000154165.5	GPR15	G protein-coupled receptor 15	Not Reported
1 ENSG00000156113.23	KCNMA1	potassium calcium-activated channel subfamily M alpha 1	Tabone, 2021 - Clinical TB compared to control
1 ENSG00000185745.10	IFIT1	interferon induced protein with tetratricopeptide repeats 1	Not Reported
1 ENSG00000211821.2	TRDV2	T cell receptor delta variable 2	Not Reported
1 ENSG00000213386.3	AC022217.2	family with sequence similarity 58, member A	Not Reported
1 ENSG00000247627.2	MTND4P12	MT-ND4 pseudogene 12	Not Reported
1 ENSG00000272398.6	CD24	CD24 molecule	Not Reported
1 ENSG00000286330.1	AL353660.1	novel transcript	Not Reported

LTBI-SI

ID - DOWN GENES	Symbol	Description	Literature review
1 ENSG00000003249.13	DBNDD1	dysbindin domain containing 1	Not Reported
1 ENSG00000005381.8	MPO	myeloperoxidase	Kaforou, 2013 - up in TB compared with LTBI
1 ENSG00000007038.11	PRSS21	serine protease 21	Not Reported
1	MMP25	matrix metallopeptidase 25	Not Reported
1 ENSG00000010704.18	HFE	homeostatic iron regulator	Not Reported
1 ENSG00000026559.14	KCNG1	potassium voltage-gated channel modifier subfamily G member 1	Not Reported
1	MYOM2	myomesin 2	Not Reported
1 ENSG00000040633.13	PHF23	PHD finger protein 23	Not Reported
1	HYAL2	hyaluronidase 2	Not Reported
1	CLTCL1	clathrin heavy chain like 1	Not Reported
1	CEACAM1	CEA cell adhesion molecule 1	Not Reported
1	CEACAM6	CEA cell adhesion molecule 6	Not Reported
1	AURKA	aurora kinase A	Not Reported
1 ENSG00000091106.19	NLRC4	NLR family CARD domain containing 4	Not Reported
1	CD200	CD200 molecule	Not Reported
1 ENSG00000092067.5	CEBPE	CCAAT enhancer binding protein epsilon	Not Reported
1	COL9A3	collagen type IX alpha 3 chain	Not Reported
1 ENSG00000100311.17	PDGFB	platelet derived growth factor subunit B	Not Reported
1	TCL1A	T cell leukemia/lymphoma 1A	Not Reported
1 ENSG00000103196.12	CRISPLD2	cysteine rich secretory protein LCCL domain containing 2	Not Reported
1	AEBP1	AE binding protein 1	Not Reported
1	ERLIN1	ER lipid raft associated 1	Not Reported
1 ENSG00000109814.12	UGDH	UDP-glucose 6-dehydrogenase	Not Reported
1	MANSC1	MANSC domain containing 1	Not Reported
1 ENSG00000111291.8	GPRC5D	G protein-coupled receptor class C group 5 member D	Not Reported
1 ENSG00000112195.9	TREML2	triggering receptor expressed on myeloid cells like 2	Not Reported
1 ENSG00000114737.15	CISH	cytokine inducible SH2 containing protein	Not Reported

1	GCA	grancalcin	Not Reported
1	WLS	Wnt ligand secretion mediator	Not Reported
1	BMP8B	bone morphogenetic protein 8b	Not Reported
1	PADI2	peptidyl arginine deiminase 2	Not Reported
1 ENSG00000117399.14	CDC20	cell division cycle 20	Not Reported
1	MMP8	matrix metallopeptidase 8	Not Reported
1 ENSG00000119121.22	TRPM6	transient receptor potential cation channel subfamily M member 6	Not Reported
1	SLC46A2	olute carrier family 46 member 2	Not Reported
1 ENSG00000120049.19	KCNIP2	potassium voltage-gated channel interacting protein 2	Not Reported
1 ENSG00000121716.20	PILRB	paired immunoglobin like type 2 receptor beta	Not Reported
1	CCR2	C-C motif chemokine receptor 2	Not Reported
1	TNFSF10	TNF superfamily member 10	Not Reported
1 ENSG00000121931.16	LRIF1	ligand dependent nuclear receptor interacting factor 1	Not Reported
1	NFE2	nuclear factor, erythroid 2	Not Reported
1	EEF1AKMT3	EEF1A lysine methyltransferase 3	Not Reported
1	MKKS	McKusick-Kaufman syndrome	Not Reported
1	AOC3	amine oxidase copper containing 3	Not Reported
1 ENSG00000132763.15	MMACHC	metabolism of cobalamin associated C	Not Reported
1	CHIT1	chitinase 1	Not Reported
1 ENSG00000133561.15	GIMAP6	GTPase, IMAP family member 6	Not Reported
1 ENSG00000134057.15	CCNB1	cyclin B1	Not Reported
1 ENSG00000134061.5	CD180	CD180 molecule	Not Reported
1 ENSG00000134827.8	TCN1	transcobalamin 1	Not Reported
1	AVIL	advillin	Not Reported
1	GPR55	G protein-coupled receptor 55	Not Reported
1	HLX	H2.0 like homeobox	Not Reported

1	PRICKLE1	prickle planar cell polarity protein 1	Not Reported
1	LPAR6	lysophosphatidic acid receptor 6	Not Reported
1 ENSG00000139998.15	RAB15	RAB15, member RAS oncogene family	Not Reported
1 ENSG00000142405.21	NLRP12	NLR family pyrin domain containing 12	Gebremedhin Gebremicael, 2019 - Down in TB compared with LTBI
1	SLC2A5	solute carrier family 2 member 5	Not Reported
1 ENSG00000142675.18	CNKSR1	connector enhancer of kinase suppressor of Ras 1	Not Reported
1	GOLPH3L	golgi phosphoprotein 3 like	Not Reported
1 ENSG00000143878.10	RHOB	ras homolog family member B	Wu LS, 2014 - LTBI versus control upregulated
1	GIN1	gypsy retrotransposon integrase 1	Not Reported
1	GNRH1	gonadotropin releasing hormone 1	Not Reported
1	LACTB2	lactamase beta 2	Not Reported
1	MS4A3	membrane spanning 4-domains A3	Not Reported
1	MS4A2	membrane spanning 4-domains A2	Not Reported
1 ENSG00000150967.18	ABCB9	ATP binding cassette subfamily B member 9	Not Reported
1 ENSG00000152213.4	ARL11	ADP ribosylation factor like GTPase 11	Not Reported
1 ENSG00000154734.15	ADAMTS1	ADAM metallopeptidase with thrombospondin type 1 motif 1	Not Reported
1 ENSG00000157551.19	KCNJ15	potassium inwardly rectifying channel subfamily J member 15	Philip Kam Weng Kwan, 2020 - Active TB vs healthy controls/IGRA negative
1 ENSG00000158715.6	SLC45A3	solute carrier family 45 member 3	Not Reported
1	PADI4	peptidyl arginine deiminase 4	Not Reported
1 ENSG00000160113.5	NR2F6	nuclear receptor subfamily 2 group F member 6	Not Reported
1 ENSG00000160285.15	LSS	lanosterol synthase	Not Reported
1	TTC9C	tetratricopeptide repeat domain 9C	Not Reported
1 ENSG00000162747.11	FCGR3B	Fc fragment of IgG receptor IIIb	Philip Kam Weng Kwan, 2020 - Exposed contact compared to control
1	TNFAIP8L2	NF alpha induced protein 8 like 2	Not Reported
1	SLC22A15	solute carrier family 22 member 15	Not Reported
1 ENSG00000163464.8	CXCR1	C-X-C motif chemokine receptor 1	Philip Kam Weng Kwan, 2020 - Exposed contact compared to control
1 ENSG00000163563.8	MNDA	myeloid cell nuclear differentiation antigen	Not Reported

1 ENSG00000163606.11	CD200R1	CD200 receptor 1	Not Reported
1	TMEM144	transmembrane protein 144	Not Reported
1 ENSG00000164403.14	SHROOM1	shroom family member 1	Not Reported
1	C9orf64	chromosome 9 open reading frame 64	Not Reported
1 ENSG00000165646.14	SLC18A2	solute carrier family 18 member A2	Not Reported
1	GPT2	glutamic--pyruvic transaminase 2	Not Reported
1	KIAA0355	KIAA0355	Not Reported
1	PLK1	polo like kinase 1	Not Reported
1	LOXHD1	lipoxygenase homology domains 1	Not Reported
1	NT5DC2	5'-nucleotidase domain containing 2	Not Reported
1	CX3CR1	C-X3-C motif chemokine receptor 1	Not Reported
1 ENSG00000169116.11	PARM1	prostate androgen-regulated mucin-like protein 1	Not Reported
1	OSCAR	osteoclast associated Ig-like receptor	Not Reported
1 ENSG00000170915.9	PAQR8	progestin and adipoQ receptor family member 8	Not Reported
1	GIMAP8	GTPase, IMAP family member 8	Not Reported
1 ENSG00000173110.8	HSPA6	heat shock protein family A (Hsp70) member 6	Not Reported
1 ENSG00000173535.14	TNFRSF10C	TNF receptor superfamily member 10c	Wang S, 2019 - Up in LTBI compared to active TB
1	CEP19	centrosomal protein 19	Not Reported
1 ENSG00000174123.11	TLR10	toll like receptor 10	Not Reported
1	CMKLR1	chemerin chemokine-like receptor 1	Not Reported
1 ENSG00000175857.8	GAPT	GRB2 binding adaptor protein, transmembrane	Not Reported
1	ZNF404	zinc finger protein 404	Not Reported
1	RMI1	RecQ mediated genome instability 1	Not Reported
1 ENSG00000179889.19	PDXDC1	pyridoxal dependent decarboxylase domain containing 1	Not Reported
1	FZD2	frizzled class receptor 2	Not Reported
1	FUT7	fucosyltransferase 7	Not Reported
1	CXCR2	C-X-C motif chemokine receptor 2	Not Reported
1	ST20	suppressor of tumorigenicity 20	Not Reported
1	P2RY13	purinergic receptor P2Y13	Not Reported
1 ENSG00000182700.5	IGIP	IgA inducing protein	Not Reported
1	TMEM121B	transmembrane protein 121B	Not Reported
1 ENSG00000183625.15	CCR3	C-C motif chemokine receptor 3	Not Reported
1	METTL7A	methyltransferase like 7A	Not Reported
1 ENSG00000185745.10	IFIT1	interferon induced protein with tetratricopeptide repeats 1	Not Reported

1 ENSG00000186205.13	MARC1	mitochondrial amidoxime reducing component 1	Not Reported
1 ENSG00000186529.16	CYP4F3	cytochrome P450 family 4 subfamily F member 3	Not Reported
1	GCNT1	glucosaminyl (N-acetyl) transferase 1	Not Reported
1 ENSG00000187554.14	TLR5	toll like receptor 5	Gebremedhin Gebremicael, 2019 - Up in TB compared with LTBI
1	PEAK3	PEAK family member 3	Not Reported
1	MIRLET7BH	MIRLET7B host gene	Not Reported
1 ENSG00000197520.10	FAM177B	family with sequence similarity 177 member B	Not Reported
1 ENSG00000198440.9	ZNF583	zinc finger protein 583	Not Reported
1	ZNF28	zinc finger protein 28	Not Reported
1	MSRB1	methionine sulfoxide reductase B1	Not Reported
1	TRAV12-2	T cell receptor alpha variable 12-2	Not Reported
1	IGHV1-2	immunoglobulin heavy variable 1-2	Not Reported
1 ENSG00000211941.3	IGHV3-11	immunoglobulin heavy variable 3-11 (gene/pseudogene)	Not Reported
1	HOMEZ	homeobox and leucine zipper encoding	Not Reported
1 ENSG00000225101.6	OR52K3P	olfactory receptor family 52 subfamily K member 3 pseudogene	Philip Kam Weng Kwan, 2020 - Active TB vs healthy controls/IGRA negative
1 ENSG00000225194.3	LINC00092	long intergenic non-protein coding RNA 92	Not Reported
1	AL590764.1	novel transcript	Not Reported
1 ENSG00000235314.1	LINC00957	long intergenic non-protein coding RNA 957	Not Reported
1 ENSG00000238113.7	LINC01410	long intergenic non-protein coding RNA 1410	Not Reported
1	IGKV1-6	immunoglobulin kappa variable 1-6	Not Reported
1	IGKV1-17	immunoglobulin kappa variable 1-17	Not Reported
1	IGKV1-16	immunoglobulin kappa variable 1-16	Not Reported
1	IGKV3-11	immunoglobulin kappa variable 3-11	Not Reported
1	IGKV1-9	immunoglobulin kappa variable 1-9	Not Reported
1	HYPK	huntingtin interacting protein K	Not Reported
1 ENSG00000244482.10	LILRA6	leukocyte immunoglobulin like receptor A6	Not Reported
1 ENSG00000250251.6	PKD1P6	polycystin 1, transient receptor potential channel interacting pseudogene 6	Not Reported
1	GPR162	G protein-coupled receptor 162	Not Reported
1 ENSG00000253239.1	IGLVI-70	mmunoglobulin lambda variable (I)-70 (pseudogene)	Not Reported

1	AP001372.2	uncharacterized LOC100287896	Not Reported
1 ENSG00000254838.5	GVINP1	GTPase, very large interferon inducible pseudogene 1	Not Reported
1	TMEM9B-AS1	TMEM9B antisense RNA 1	Not Reported
1	AL139020.1	novel transcript, antisense to TCL1A	Not Reported
1 ENSG00000260528.5	FAM157C	amily with sequence similarity 157 member C	Philip Kam Weng Kwan, 2020 - Exposed contact compared to control
1 ENSG00000260828.1	HMGB3P32	high mobility group box 3 pseudogene 32	Not Reported
1	ZNNT1	ZNF706 neighboring transcript 1	Not Reported
1 ENSG00000263961.8	RHEX	regulator of hemoglobinization and erythroid cell expansion	Not Reported
1	AL627309.7	novel pseudogene	Not Reported
1	CD24	CD24 molecule	Not Reported
1	AL590560.3	novel transcript, antisense to VSIG8	Not Reported
1	AP005329.3	novel transcript	Not Reported
1	AC006033.2	novel transcript	Not Reported
1	AL034397.3	novel transcript, antisense VSIG4	Not Reported
1	ZNF2	zinc finger protein 2	Not Reported
1	HERC2P2	hect domain and RLD 2	Not Reported
1	SSTR3	somatostatin receptor 3	Not Reported
1	CCDC163	coiled-coil domain containing 163	Not Reported
1 ENSG00000280832.1	GSEC	G-quadruplex forming sequence containing lncRNA	Not Reported
1 ENSG00000281162.2	LINC01127	long intergenic non-protein coding RNA 1127	Not Reported

ID - UP GENES	Symbol	Description	Literature review
ENSG00000061656.10	SPAG4	sperm associated antigen 4	Not Reported
1 ENSG00000076706.17	MCAM	melanoma cell adhesion molecule	Not Reported
1 ENSG00000081041.9	CXCL2	C-X-C motif chemokine ligand 2	Wu LS, 2014 - LTBI versus control upregulated
1 ENSG00000085840.13	ORC1	origin recognition complex subunit 1	Not Reported
1 ENSG00000088899.15	LZTS3	leucine zipper tumor suppressor family member 3	Not Reported
1 ENSG00000095794.19	CREM	cAMP responsive element modulator	Not Reported
1 ENSG00000100003.18	SEC14L2	SEC14 like lipid binding 2	Not Reported
1 ENSG00000100906.10	NFKBIA	NFKB inhibitor alpha	Lee SW, 2016 - Down LTBI conpared to control and active TB
1 ENSG00000101665.9	SMAD7	SMAD family member 7	Not Reported
1 ENSG00000102678.7	FGF9	fibroblast growth factor 9	Not Reported
1 ENSG00000104660.19	LEPROTL1	leptin receptor overlapping transcript like 1	Not Reported
1 ENSG00000104671.8	DCTN6	dynactin subunit 6	Not Reported
1 ENSG00000104856.14	RELB	RELB proto-oncogene, NF-kB subunit	Not Reported
1 ENSG00000104951.16	IL4I1	interleukin 4 induced 1	Not Reported
1 ENSG00000108821.13	COL1A1	collagen type I alpha 1 chain	Not Reported
1 ENSG00000109929.10	SC5D	sterol-C5-desaturase	Not Reported
1 ENSG00000112096.18	SOD2	superoxide dismutase 2	Not Reported
1 ENSG00000112137.17	PHACTR1	phosphatase and actin regulator 1	Not Reported
1 ENSG00000113448.19	PDE4D	phosphodiesterase 4D	Not Reported
1 ENSG00000114315.4	HES1	hes family bHLH transcription factor 1	Not Reported
1 ENSG00000115009.13	CCL20	C-C motif chemokine ligand 20	Not Reported
1 ENSG00000116285.13	ERRFI1	ERBB receptor feedback inhibitor 1	Not Reported
1 ENSG00000116717.13	GADD45A	growth arrest and DNA damage inducible alpha	Not Reported
1 ENSG00000117519.16	CNN3	calponin 3	Not Reported
1 ENSG00000118503.15	TNFAIP3	TNF alpha induced protein 3	Not Reported
1 ENSG00000118985.16	ELL2	elongation factor for RNA polymerase II 2	Not Reported
1 ENSG00000119508.18	NR4A3	nuclear receptor subfamily 4 group A member 3	Not Reported

1 ENSG00000119986.7	AVPI1	arginine vasopressin induced 1	Not Reported
1 ENSG00000120875.9	DUSP4	dual specificity phosphatase 4	Not Reported
1 ENSG00000121797.10	CCRL2	C-C motif chemokine receptor like 2	Not Reported
1 ENSG00000123342.16	MMP19	matrix metallopeptidase 19	Not Reported
1 ENSG00000123685.9	BATF3	basic leucine zipper ATF-like transcription factor 3	Not Reported
1 ENSG00000124145.6	SDC4	syndecan 4	Not Reported
1 ENSG00000124466.9	LYPD3	LY6/PLAUR domain containing 3	Not Reported
1 ENSG00000125084.12	WNT1	Wnt family member 1	Not Reported
1 ENSG00000125319.14	HROB	homologous recombination factor with OB-fold	Not Reported
1 ENSG00000125657.5	TNFSF9	TNF superfamily member 9	Not Reported
1 ENSG00000125726.11	CD70	CD70 molecule	Not Reported
1 ENSG00000127081.14	ZNF484	zinc finger protein 484	Not Reported
1 ENSG00000130340.16	SNX9	sorting nexin 9	Not Reported
1 ENSG00000130844.18	ZNF331	zinc finger protein 331	Not Reported
1 ENSG00000132819.17	RBM38	RNA binding motif protein 38	Not Reported
1 ENSG00000134107.5	BHLHE40	basic helix-loop-helix family member e40	Not Reported
1 ENSG00000135047.15	CTSL	cathepsin L	Not Reported
1 ENSG00000135114.12	OASL	2'-5'-oligoadenylate synthetase like	Philip Kam Weng Kwan, 2020 - Exposed contact compared to control
1 ENSG00000136244.12	IL6	interleukin 6	Not Reported
1 ENSG00000136689.18	IL1RN	interleukin 1 receptor antagonist	Philip Kam Weng Kwan, 2020- Active TB vs healthy controls/IGRA negative<br><br>Lee SW, 2016 - Down LTBI compared to active TB
1 ENSG00000137507.11	LRRC32	leucine rich repeat containing 32	Philip Kam Weng Kwan, 2020 - Exposed contact compared to control
1 ENSG00000138641.18	HERC3	HECT and RLD domain containing E3 ubiquitin protein ligase 3	Not Reported
1 ENSG00000139289.13	PHLDA1	pleckstrin homology like domain family A member 1 [	Not Reported
1 ENSG00000139572.4	GPR84	G protein-coupled receptor 84	Not Reported
1 ENSG00000143333.7	RGS16	regulator of G protein signaling 16	Not Reported
1 ENSG00000144115.16	THNSL2	threonine synthase like 2	Not Reported

1 ENSG00000145911.6	N4BP3	NEDD4 binding protein 3	Not Reported
1 ENSG00000148344.11	PTGES	prostaglandin E synthase	Not Reported
1 ENSG00000152409.9	JMY	junction mediating and regulatory protein, p53 cofactor	Not Reported
1 ENSG00000153234.14	NR4A2	nuclear receptor subfamily 4 group A member 2	Not Reported
1 ENSG00000154099.18	DNAAF1	dynein axonemal assembly factor 1	Not Reported
1 ENSG00000155380.11	SLC16A1	solute carrier family 16 member 1	Not Reported
1 ENSG00000158747.14	NBL1	NBL1, DAN family BMP antagonist	Not Reported
1 ENSG00000160789.20	LMNA	lamin A/C	Not Reported
1 ENSG00000162924.14	REL	REL proto-oncogene, NF-kB subunit	Not Reported
1 ENSG00000163600.12	ICOS	inducible T cell costimulator	Not Reported
1 ENSG00000163661.4	PTX3	pentraxin 3	Not Reported
1 ENSG00000163734.4	CXCL3	C-X-C motif chemokine ligand 3	Not Reported
1 ENSG00000164236.12	ANKRD33B	ankyrin repeat domain 33B	Not Reported
1 ENSG00000165410.15	CFL2	cofilin 2	Not Reported
1 ENSG00000165474.8	GJB2	gap junction protein beta 2	Not Reported
1 ENSG00000166165.13	CKB	creatine kinase B	Not Reported
1 ENSG00000166451.13	CENPN	centromere protein N	Not Reported
1 ENSG00000166592.12	RRAD	RRAD, Ras related glycolysis inhibitor and calcium channel regulator	Not Reported
1 ENSG00000166920.12	C15orf48	chromosome 15 open reading frame 48 [	Not Reported
1 ENSG00000167034.10	NKX3-1	NK3 homeobox 1	Not Reported
1 ENSG00000167618.10	LAIR2	leukocyte associated immunoglobulin like receptor 2	Not Reported
1 ENSG00000168994.13	PXDC1	PX domain containing 1	Not Reported
1 ENSG00000170889.14	RPS9	ribosomal protein S9	Not Reported
1 ENSG00000170989.9	S1PR1	sphingosine-1-phosphate receptor 1	Not Reported
1 ENSG00000171617.14	ENC1	ectodermal-neural cortex 1	Not Reported
1 ENSG00000172602.11	RND1	Rho family GTPase 1	Not Reported
1 ENSG00000173451.7	THAP2	THAP domain containing 2	Not Reported

1 ENSG00000176105.14	YES1	YES proto-oncogene 1, Src family tyrosine kinase	Not Reported
1 ENSG00000176490.5	DIRAS1	DIRAS family GTPase 1	Not Reported
1 ENSG00000176845.13	METRNL	meteorin like, glial cell differentiation regulator	Not Reported
1 ENSG00000177374.13	HIC1	HIC ZBTB transcriptional repressor 1	Not Reported
1 ENSG00000178803.12	ADORA2A-AS1	ADORA2A antisense RNA 1	Not Reported
1 ENSG00000180611.7	MB21D2	Mab-21 domain containing 2	Not Reported
1 ENSG00000182687.4	GALR2	galanin receptor 2	Not Reported
1 ENSG00000182853.12	VMO1	vitelline membrane outer layer 1 homolog	Not Reported
1 ENSG00000183484.12	GPR132	G protein-coupled receptor 132	Not Reported
1 ENSG00000183508.5	TENT5C	terminal nucleotidyltransferase 5C	Not Reported
1 ENSG00000183813.7	CCR4	C-C motif chemokine receptor 4	Not Reported
1 ENSG00000184163.3	C1QTNF12	C1q and TNF related 12	Not Reported
1 ENSG00000184545.11	DUSP8	dual specificity phosphatase 8	Not Reported
1 ENSG00000184588.18	PDE4B	phosphodiesterase 4B	Not Reported
1 ENSG00000188290.10	HES4	hes family bHLH transcription factor 4	Not Reported
1 ENSG00000189057.11	FAM111B	family with sequence similarity 111 member B	Not Reported
1 ENSG00000197122.11	ENSG00000197122	SRC proto-oncogene, non-receptor tyrosine kinase	Not Reported
1 ENSG00000197632.9	SERPINB2	serpin family B member 2	Not Reported
1 ENSG00000197860.10	SGTB	small glutamine rich tetratricopeptide repeat containing beta	Not Reported
1 ENSG00000198369.10	SPRED2	sprouty related EVH1 domain containing 2	Not Reported
1 ENSG00000204001.9	LCN8	lipocalin 8	Tabone, 2021 - Up Incipient TB compared to control
1 ENSG00000204186.10	ZDBF2	zinc finger DBF-type containing 2	Not Reported
1 ENSG00000211665.3	IGLV3-16	immunoglobulin lambda variable 3-16	Not Reported
1 ENSG00000218018.3	AL109955.1	novel transcript	Not Reported
1 ENSG00000220749.4	RPL21P28	ribosomal protein L21 pseudogene 28	Not Reported
1 ENSG00000222009.8	BTBD19	BTB domain containing 19	Not Reported
1 ENSG00000230002.3	ALMS1-IT1	ALMS1 intronic transcript 1	Not Reported
1 ENSG00000234883.6	MIR155HG	MIR155 host gene	Not Reported
1 ENSG00000237973.1	MTCO1P12	MT-CO1 pseudogene 12	Not Reported
1 ENSG00000237989.1	LINC01679	long intergenic non-protein coding RNA 1679	Not Reported
1 ENSG00000238039.1	AC244197.2	novel transcript	Not Reported
1 ENSG00000250321.1	AC079140.2	pseudogene similar to part of ribosomal protein S17 (RPS17)	Not Reported
1 ENSG00000260317.1	AC009812.4	novel transcript	Not Reported
1 ENSG00000260708.1	AL118516.1	novel transcript, antisense to TBC1D22A	Not Reported

1	ENSG00000266709.1	AC005224.3	novel transcript	Not Reported
1	ENSG00000267365.1	KCNJ2-AS1	KCNJ2 antisense RNA 1	Not Reported
1	ENSG00000270681.1	AC095055.1	novel transcript, antisense to SH3D19	Not Reported
1	ENSG00000271781.1	AC026740.1	novel transcript, antisense to TPPP	Not Reported
1	ENSG00000273951.1	AL031667.3	novel transcript, sense intronic to CHD6	Not Reported
1	ENSG00000274286.2	ADRA2B	adrenoceptor alpha 2B	Not Reported
1	ENSG00000274677.1	AC040169.3	novel transcript, sense intronic to MBTPS1	Not Reported
1	ENSG00000275302.2	CCL4	C-C motif chemokine ligand 4	Gebremedhin Gebremicael, 2019 - Down in TB compared with LTBI
1	ENSG00000276070.5	CCL4L2	C-C motif chemokine ligand 4 like 2 [	Not Reported
1	ENSG00000277632.2	CCL3	C-C motif chemokine ligand 3	Not Reported
1	ENSG00000278356.1	AC005911.1	novel transcript	Not Reported
1	ENSG00000283199.3	C13orf46	chromosome 13 open reading frame 46	Not Reported

LTBI-LI

ID - DOWN GENES	Symbol	Description	Literature review
1 ENSG00000038945.15	**MSR1**	macrophage scavenger receptor 1	Not Reported
1 ENSG00000047634.15	SCML1	Scm polycomb group protein like 1	Not Reported
1 ENSG00000112137.17	PHACTR1	phosphatase and actin regulator 1	Not Reported
1 ENSG00000114315.4	HES1	hes family bHLH transcription factor 1	Not Reported
1 ENSG00000116016.14	EPAS1	endothelial PAS domain protein 1	Not Reported
1 ENSG00000116285.13	ERRFI1	ERBB receptor feedback inhibitor 1	Not Reported
1 ENSG00000117519.16	CNN3	calponin 3	Not Reported
1 ENSG00000119508.18	NR4A3	nuclear receptor subfamily 4 group A member 3	Not Reported
1 ENSG00000119986.7	AVPI1	arginine vasopressin induced 1	Not Reported
1 ENSG00000135114.12	OASL	2'-5'-oligoadenylate synthetase like	Philip Kam Weng Kwan, 2020- Exposed contact compared to control
1 ENSG00000144115.16	THNSL2	threonine synthase like 2	Not Reported
1 ENSG00000144290.17	SLC4A10	solute carrier family 4 member 10	Not Reported
1 ENSG00000167034.10	NKX3-1	NK3 homeobox 1	Not Reported
1 ENSG00000167618.10	LAIR2	leukocyte associated immunoglobulin like receptor 2	Not Reported
1 ENSG00000168994.13	PXDC1	PX domain containing 1	Not Reported
1 ENSG00000170889.14	RPS9	ribosomal protein S9	Not Reported
1 ENSG00000171617.14	ENC1	ectodermal-neural cortex 1	Not Reported
1 ENSG00000176749.9	CDK5R1	cyclin dependent kinase 5 regulatory subunit 1	Not Reported
1 ENSG00000177374.13	HIC1	HIC ZBTB transcriptional repressor 1	Lee SW, 2016 - Down TB compared to control

1 ENSG00000182853.12	VMO1	vitelline membrane outer layer 1 homolog	Not Reported
1 ENSG00000183813.7	CCR4	C-C motif chemokine receptor 4	Not Reported
1 ENSG00000188290.10	HES4	hes family bHLH transcription factor 4	Not Reported
1 ENSG00000196126.11	HLA-DRB1	major histocompatibility complex, class II, DR beta 1	Not Reported
1 ENSG00000198369.10	SPRED2	sprouty related EVH1 domain containing 2	Not Reported
1 ENSG00000204001.9	LCN8	lipocalin 8	Tabone, 2021 - Incipient TB compared to control
1 ENSG00000205426.10	KRT81	keratin 81	Not Reported
1 ENSG00000211821.2	TRDV2	T cell receptor delta variable 2	Not Reported
1 ENSG00000220793.5	RPL21P119	ribosomal protein L21 pseudogene 119	Not Reported
1 ENSG00000237989.1	LINC01679	long intergenic non-protein coding RNA 1679	Not Reported
1 ENSG00000250321.1	AC079140.2	pseudogene similar to part of ribosomal protein S17	Not Reported
1 ENSG00000274677.1	AC040169.3	novel transcript, sense intronic to MBTPS1	Not Reported
1 ENSG00000275302.2	CCL4	C-C motif chemokine ligand 4	Not Reported
1 ENSG00000283199.3	C13orf46	chromosome 13 open reading frame 46	Not Reported

ID - UP GENES	Symbol	Description	Literature review
1 ENSG00000003249.13	DBNDD1	dysbindin domain containing 1	Not Reported
1 ENSG00000007038.11	PRSS21	serine protease 21	Not Reported
1 ENSG00000008516.17	MMP25	matrix metallopeptidase 25	Not Reported
1 ENSG00000060140.9	STYK1	serine/threonine/tyrosine kinase 1	Not Reported
1 ENSG00000087586.17	AURKA	aurora kinase A	Not Reported
1 ENSG00000092758.18	COL9A3	collagen type IX alpha 3 chain	Not Reported
1 ENSG00000099958.14	DERL3	derlin 3	Not Reported
1 ENSG00000100311.17	PDGFB	platelet derived growth factor subunit B	Not Reported
1 ENSG00000100721.11	TCL1A	T cell leukemia/lymphoma 1A [	Not Reported
1 ENSG00000110777.12	POU2AF1	OU class 2 homeobox associating factor 1	Not Reported
1 ENSG00000111261.14	MANSC1	MANSC domain containing 1	Not Reported
1 ENSG00000111291.8	GPRC5D	G protein-coupled receptor class C group 5 member D	Not Reported
1 ENSG00000115884.11	SDC1	syndecan 1	Not Reported
1 ENSG00000116729.14	WLS	Wnt ligand secretion mediator	Not Reported
1 ENSG00000116985.12	BMP8B	bone morphogenetic protein 8b	Not Reported
1 ENSG00000117399.14	CDC20	cell division cycle 20	Not Reported
1 ENSG00000120049.19	KCNIP2	potassium voltage-gated channel interacting protein 2	Not Reported
1 ENSG00000130812.10	ANGPTL6	angiopoietin like 6	Not Reported
1 ENSG00000131400.8	NAPSA	napsin A aspartic peptidase [	Not Reported

1 ENSG00000132465.11	JCHAIN	joining chain of multimeric IgA and IgM	Not Reported
1 ENSG00000133328.4	PLAAT2	phospholipase A and acyltransferase 2	Not Reported
1 ENSG00000134057.15	CCNB1	cyclin B1	Not Reported
1 ENSG00000134061.5	CD180	CD180 molecule	Not Reported
1 ENSG00000135898.10	GPR55	G protein-coupled receptor 55	Not Reported
1 ENSG00000135916.16	ITM2C	integral membrane protein 2C	Not Reported
1 ENSG00000142583.18	SLC2A5	solute carrier family 2 member 5	Not Reported
1 ENSG00000142675.18	CNKSR1	connector enhancer of kinase suppressor of Ras 1	Not Reported
1 ENSG00000150967.18	ABCB9	ATP binding cassette subfamily B member 9	Not Reported
1 ENSG00000159339.13	PADI4	peptidyl arginine deiminase 4	Not Reported
1 ENSG00000164124.11	TMEM144	transmembrane protein 144	Not Reported
1 ENSG00000164403.14	SHROOM1	shroom family member 1	Not Reported
1 ENSG00000166123.14	GPT2	glutamic--pyruvic transaminase 2	Not Reported
1 ENSG00000166851.15	PLK1	polo like kinase 1	Not Reported
1 ENSG00000168268.11	NT5DC2	5'-nucleotidase domain containing 2	Not Reported
1 ENSG00000169116.11	PARM1	prostate androgen-regulated mucin-like protein 1	Not Reported
1 ENSG00000170909.13	OSCAR	osteoclast associated Ig-like receptor	Not Reported
1 ENSG00000178445.9	GLDC	glycine decarboxylase	Not Reported
1 ENSG00000180535.3	BHLHA15	basic helix-loop-helix family member a15	Not Reported
1 ENSG00000180549.7	FUT7	fucosyltransferase 7	Not Reported
1 ENSG00000183010.17	PYCR1	pyrroline-5-carboxylate reductase 1	Not Reported
1 ENSG00000185432.12	METTL7A	methyltransferase like 7A	Not Reported
1 ENSG00000186529.16	CYP4F3	cytochrome P450 family 4 subfamily F member 3	Not Reported

1 ENSG00000187554.14	TLR5	toll like receptor 5	Simon Blankley, 2016 - Up active tb compared to control<br><br>Gebremedhin Gebremicael, 2019 -lower expressioninTST+ comparedtoTST-, higher expression un TB compared to TST+ and TST-
1 ENSG00000211597.2	IGKJ1	immunoglobulin kappa joining 1	Not Reported
1 ENSG00000211632.4	IGKV3D-11	immunoglobulin kappa variable 3D-11	Not Reported
1 ENSG00000211640.4	IGLV6-57	immunoglobulin lambda variable 6-57	Not Reported
1 ENSG00000211659.2	IGLV3-25	immunoglobulin lambda variable 3-25	Not Reported
1 ENSG00000211668.2	IGLV2-11	immunoglobulin lambda variable 2-11	Not Reported
1 ENSG00000211679.2	IGLC3	immunoglobulin lambda constant 3 (Kern-Oz+ marker)	Not Reported
1 ENSG00000211892.4	IGHG4	immunoglobulin heavy constant gamma 4	Not Reported
1 ENSG00000211898.7	IGHD	immunoglobulin heavy constant delta [	Not Reported
1 ENSG00000211934.3	IGHV1-2	immunoglobulin heavy variable 1-2	Not Reported
1 ENSG00000211935.3	IGHV1-3	immunoglobulin heavy variable 1-3 [	Not Reported
1 ENSG00000211937.3	IGHV2-5	immunoglobulin heavy variable 2-5	Not Reported
1 ENSG00000211941.3	IGHV3-11	immunoglobulin heavy variable 3-11	Not Reported
1 ENSG00000222037.5	IGLC6	immunoglobulin lambda constant 6 (gene/pseudogene)	Not Reported
1 ENSG00000224041.3	IGKV3D-15	immunoglobulin kappa variable 3D-15 (gene/pseudogene)	Not Reported
1 ENSG00000228427.3	AL590764.1	novel transcript	Not Reported
1 ENSG00000230006.7	ANKRD36BP 2	ankyrin repeat domain 36B pseudogene 2	Not Reported
1 ENSG00000237649.8	KIFC1	kinesin family member C1	Not Reported
1 ENSG00000239571.1	IGKV2D-30	immunoglobulin kappa variable 2D-30	Not Reported

1 ENSG00000239855.1	IGKV1-6	immunoglobulin kappa variable 1-6	Not Reported
1 ENSG00000239951.1	IGKV3-20	immunoglobulin kappa variable 3-20	Not Reported
1 ENSG00000239975.2	IGKV1D-33	immunoglobulin kappa variable 1D-33	Not Reported
1 ENSG00000240382.3	IGKV1-17	immunoglobulin kappa variable 1-17 [	Not Reported
1 ENSG00000240864.3	IGKV1-16	immunoglobulin kappa variable 1-16	Not Reported
1 ENSG00000241351.3	IGKV3-11	immunoglobulin kappa variable 3-11	Not Reported
1 ENSG00000241755.1	IGKV1-9	immunoglobulin kappa variable 1-9	Not Reported
1 ENSG00000242076.2	IGKV1-33	immunoglobulin kappa variable 1-33	Not Reported
1 ENSG00000242371.1	IGKV1-39	mmunoglobulin kappa variable 1-39	Not Reported
1 ENSG00000242534.2	IGKV2D-28	immunoglobulin kappa variable 2D-28	Not Reported
1 ENSG00000243238.1	IGKV2-30	immunoglobulin kappa variable 2-30	Not Reported
1 ENSG00000243466.1	IGKV1-5	immunoglobulin kappa variable 1-5	Not Reported
1 ENSG00000244116.3	IGKV2-28	immunoglobulin kappa variable 2-28	Not Reported
1 ENSG00000244437.1	IGKV3-15	immunoglobulin kappa variable 3-15	Not Reported
1 ENSG00000244575.3	IGKV1-27	immunoglobulin kappa variable 1-27	Not Reported
1 ENSG00000251546.1	IGKV1D-39	immunoglobulin kappa variable 1D-39	Not Reported
1 ENSG00000253239.1	IGLVI-70	immunoglobulin lambda variable (I)-70 (pseudogene)	Not Reported
1 ENSG00000254709.8	IGLL5	immunoglobulin lambda like polypeptide 5	Not Reported
1 ENSG00000257275.6	AL139020.1	novel transcript, antisense to TCL1A	Not Reported
1 ENSG00000258572.1	AL133467.1	novel transcript	Not Reported
1 ENSG00000260528.5	FAM157C	family with sequence similarity 157 member C	Philip Kam Weng Kwan, 2020 - Exposed contact compared to control
1 ENSG00000269981.1	AL627309.7	novel pseudogene	Not Reported
1 ENSG00000278196.3	GLV2-8	immunoglobulin lambda variable 2-8 [	Not Reported

NI-SI

ID - UP GENES	Symbol	Description	Literature review
1 ENSG00000094804.12	CDC6	cell division cycle 6	Not Reported
1 ENSG00000104267.10	CA2	carbonic anhydrase 2	Not Reported
1 ENSG00000109805.10	NCAPG	non-SMC condensin I complex subunit G	Not Reported
1 ENSG00000111206.12	FOXM1	forkhead box M1	Not Reported
1 ENSG00000117724.13	CENPF	centromere protein F	Not Reported
1 ENSG00000135476.11	ESPL1	extra spindle pole bodies like 1, separase	Not Reported
1 ENSG00000145386.10	CCNA2	cyclin A2	Not Reported
1 ENSG00000146670.10	CDCA5	cell division cycle associated 5	Not Reported
1 ENSG00000150681.10	RGS18	regulator of G protein signaling 18	Not Reported
1 ENSG00000158406.4	H4C8	H4 clustered histone 8	Not Reported
1 ENSG00000161888.11	SPC24	SPC24 component of NDC80 kinetochore complex	Not Reported
1 ENSG00000167513.9	CDT1	chromatin licensing and DNA replication factor 1	Not Reported
1 ENSG00000171848.15	RRM2	ribonucleotide reductase regulatory subunit M2	Not Reported
1 ENSG00000175746.6	C15orf54	chromosome 15 open reading frame 54 (putative)	Not Reported
1 ENSG00000187699.10	C2orf88	chromosome 2 open reading frame 88	Not Reported
1 ENSG00000198888.2	MT-ND1	mitochondrially encoded NADH:ubiquinone oxidoreductase core subunit 1	Not Reported
1 ENSG00000205426.10	KRT81	keratin 81	Not Reported
1 ENSG00000211638.2	IGLV8-61	immunoglobulin lambda variable 8-61	Not Reported
1 ENSG00000211639.2	IGLV4-60	immunoglobulin lambda variable 4-6	Not Reported
1 ENSG00000211648.2	IGLV1-47	immunoglobulin lambda variable 1-47	Not Reported
1 ENSG00000211673.2	IGLV3-1	immunoglobulin lambda variable 3-1	Not Reported
1 ENSG00000211676.2	IGLJ2	immunoglobulin lambda joining 2	Not Reported
1 ENSG00000220793.5	RPL21P119	ribosomal protein L21 pseudogene 119	Not Reported
1 ENSG00000227165.9	WDR11-AS1	WDR11 antisense RNA 1	Not Reported

1 ENSG00000229344.1	MTCO2P12	MT-CO2 pseudogene 12	Not Reported
1 ENSG00000233968.7	AL157895.2	novel transcript, antisense to C10orf112	Not Reported
1 ENSG00000234618.1	RPSAP9	ribosomal protein SA pseudogene 9	Not Reported
1 ENSG00000235065.1	RPL24P2	ribosomal protein L24 pseudogene 2	Not Reported
1 ENSG00000238201.1	AC114752.2	novel transcript	Not Reported
1 ENSG00000277075.2	H2AC8	H2A clustered histone 8	Not Reported
1 ENSG00000280079.1	AC011447.7	TEC	Not Reported

ID - DOWN GENES	Symbol	Description	Literature review
1 ENSG00000107317.13	PTGDS	prostaglandin D2 synthase	Not Reported
1 ENSG00000109956.13	B3GAT1	beta-1,3-glucuronyltransferase 1	Not Reported
1 ENSG00000147234.10	FRMPD3	FERM and PDZ domain containing 3	Not Reported
1	ITGAD	integrin subunit alpha D	Not Reported
1	KLF13	Kruppel like factor 13	Not Reported
1 ENSG00000173930.9	SLCO4C1	solute carrier organic anion transporter family member 4C1	Not Reported
1	ZNF683	zinc finger protein 683	Not Reported
1 ENSG00000178537.10	SLC25A20	solute carrier family 25 member 20	Not Reported
1 ENSG00000181036.14	FCRL6	Fc receptor like 6	Not Reported
1 ENSG00000183542.5	KLRC4	killer cell lectin like receptor C4 [	Not Reported
1 ENSG00000197057.9	DTHD1	death domain containing 1	Not Reported
1 ENSG00000204525.16	HLA-C	major histocompatibility complex, class I, C	Not Reported
1 ENSG00000237604.1	AP001056.1	novel transcript	Not Reported
1 ENSG00000255441.1	AC008750.2	novel transcript, antisense to SIGLEC10	Not Reported
1 ENSG00000278420.1	MIR6819	microRNA 6819	Not Reported

NI-LI

ID - UP GENES	Symbol	Description	Literature review
ENSG00000010704.18	HFE	homeostatic iron regulator	Not Reported
1 ENSG00000040633.13	PHF23	PHD finger protein 23	Not Reported
1 ENSG00000068001.14	HYAL2	hyaluronidase 2	Not Reported
1 ENSG00000070371.16	CLTCL1	clathrin heavy chain like 1	Not Reported
1 ENSG00000103196.12	CRISPLD2	cysteine rich secretory protein LCCL domain containing 2	Not Reported
1 ENSG00000106624.11	AEBP1	AE binding protein 1	Not Reported
1 ENSG00000107317.13	PTGDS	prostaglandin D2 synthase	Not Reported
1 ENSG00000107566.14	ERLIN1	ER lipid raft associated 1	Not Reported
1 ENSG00000109814.12	UGDH	UDP-glucose 6-dehydrogenase	Not Reported
1 ENSG00000109956.13	B3GAT1	beta-1,3-glucuronyltransferase 1	Not Reported
1 ENSG00000112195.9	TREML2	triggering receptor expressed on myeloid cells like 2 [	Not Reported
1 ENSG00000114737.15	CISH	cytokine inducible SH2 containing protein	Not Reported
1 ENSG00000117115.13	PADI2	peptidyl arginine deiminase 2	Not Reported
1 ENSG00000119121.22	TRPM6	transient receptor potential cation channel subfamily M member 6	Not Reported
1 ENSG00000119457.8	SLC46A2	solute carrier family 46 member 2	Not Reported
1 ENSG00000120093.11	HOXB3	homeobox B3	Not Reported
1 ENSG00000121716.20	PILRB	paired immunoglobin like type 2 receptor beta	Not Reported
1 ENSG00000121931.16	LRIF1	ligand dependent nuclear receptor interacting factor 1	Not Reported
1 ENSG00000123427.17	EEF1AKMT3	EEF1A lysine methyltransferase 3	Not Reported
1 ENSG00000131471.7	AOC3	amine oxidase copper containing 3	Not Reported
1 ENSG00000132763.15	MMACHC	metabolism of cobalamin associated C	Not Reported
1 ENSG00000133561.15	GIMAP6	GTPase, IMAP family member 6	Not Reported
1 ENSG00000135407.10	AVIL	advillin	Not Reported
1 ENSG00000139679.15	LPAR6	lysophosphatidic acid receptor 6	Not Reported
1 ENSG00000139998.15	RAB15	RAB15, member RAS oncogene family	Not Reported
1 ENSG00000142173.15	COL6A2	collagen type VI alpha 2 chain	Not Reported
1 ENSG00000143878.10	RHOB	ras homolog family member B	Wu LS, 2014 - LTBI versus control upregulated
1 ENSG00000145723.17	GIN1	gypsy retrotransposon integrase 1	Not Reported
1 ENSG00000147234.10	FRMPD3	FERM and PDZ domain containing 3	Not Reported
1 ENSG00000147437.10	GNRH1	gonadotropin releasing hormone 1	Not Reported
1 ENSG00000147592.9	LACTB2	lactamase beta 2	Not Reported

1 ENSG00000149534.9	MS4A2	membrane spanning 4-domains A2	Not Reported
1 ENSG00000154734.15	ADAMTS1	ADAM metallopeptidase with thrombospondin type 1 motif 1 [	Not Reported
1 ENSG00000156886.12	ITGAD	integrin subunit alpha D	Not Reported
1 ENSG00000158715.6	SLC45A3	solute carrier family 45 member 3	Not Reported
1 ENSG00000160113.5	NR2F6	nuclear receptor subfamily 2 group F member 6	Not Reported
1 ENSG00000160285.15	LSS	lanosterol synthase	Not Reported
1 ENSG00000160318.6	CLDND2	claudin domain containing 2	Not Reported
1 ENSG00000162222.14	TTC9C	tetratricopeptide repeat domain 9C	Not Reported
1 ENSG00000163393.13	SLC22A15	solute carrier family 22 member 15	Not Reported
1 ENSG00000163464.8	CXCR1	C-X-C motif chemokine receptor 1	Philip Kam Weng Kwan, 2020 - Exposed contact compared to control
1 ENSG00000163606.11	CD200R1	CD200 receptor 1	Not Reported
1 ENSG00000165118.15	C9orf64	chromosome 9 open reading frame 64	Not Reported
1 ENSG00000165646.14	SLC18A2	solute carrier family 18 member A2	Not Reported
1 ENSG00000166398.13	KIAA0355	KIAA0355	Not Reported
1 ENSG00000167210.17	LOXHD1	lipoxygenase homology domains 1	Not Reported
1 ENSG00000168329.13	CX3CR1	C-X3-C motif chemokine receptor 1	Not Reported
1 ENSG00000169926.11	KLF13	Kruppel like factor 13	Not Reported
1 ENSG00000171115.4	GIMAP8	GTPase, IMAP family member 8	Not Reported
1 ENSG00000173917.10	HOXB2	homeobox B2	Not Reported
1 ENSG00000173930.9	SLCO4C1	solute carrier organic anion transporter family member 4C1	Not Reported
1 ENSG00000174600.14	CMKLR1	chemerin chemokine-like receptor 1	Not Reported
1 ENSG00000176083.17	ZNF683	zinc finger protein 683	Not Reported
1 ENSG00000176222.9	ZNF404	zinc finger protein 404	Not Reported
1 ENSG00000178162.8	FAR2P2	fatty acyl-CoA reductase 2 pseudogene 2	Not Reported
1 ENSG00000178537.10	SLC25A20	solute carrier family 25 member 20	Not Reported
1 ENSG00000179889.19	PDXDC1	pyridoxal dependent decarboxylase domain containing 1	Not Reported

1 ENSG00000180340.7	FZD2	frizzled class receptor 2	Not Reported
1 ENSG00000180953.11	ST20	suppressor of tumorigenicity 20	Not Reported
1 ENSG00000181036.14	FCRL6	Fc receptor like 6	Not Reported
1 ENSG00000182700.5	IGIP	IgA inducing protein	Not Reported
1 ENSG00000183542.5	KLRC4	killer cell lectin like receptor C4	Not Reported
1 ENSG00000183625.15	CCR3	C-C motif chemokine receptor 3	Not Reported
1 ENSG00000183734.4	ASCL2	achaete-scute family bHLH transcription factor 2	Not Reported
1 ENSG00000197057.9	DTHD1	death domain containing 1	Not Reported
1 ENSG00000197182.14	MIRLET7BH	MIRLET7B host gene	Not Reported
1 ENSG00000197520.10	FAM177B	family with sequence similarity 177 member B	Not Reported
1 ENSG00000198440.9	ZNF583	zinc finger protein 583	Not Reported
1 ENSG00000198538.11	ZNF28	zinc finger protein 28	Not Reported
1 ENSG00000198736.11	MSRB1	methionine sulfoxide reductase B1	Philip Kam Weng Kwan, 2020 - Exposed contact compared to control
1 ENSG00000204525.16	HLA-C	major histocompatibility complex, class I, C	Not Reported
1 ENSG00000211789.2	TRAV12-2	T cell receptor alpha variable 12-2	Not Reported
1 ENSG00000215271.8	HOMEZ	homeobox and leucine zipper encoding	Not Reported
1 ENSG00000225101.6	OR52K3P	olfactory receptor family 52 subfamily K member 3 pseudogene	Philip Kam Weng Kwan, 2020 - Active TB vs healthy controls/IGRA negative
1 ENSG00000225194.3	LINC00092	long intergenic non-protein coding RNA 92	Not Reported
1 ENSG00000235314.1	LINC00957	long intergenic non-protein coding RNA 957	Not Reported
1 ENSG00000238113.7	LINC01410	long intergenic non-protein coding RNA 1410	Not Reported
1 ENSG00000242028.6	HYPK	huntingtin interacting protein K	Not Reported
1 ENSG00000244482.10	LILRA6	leukocyte immunoglobulin like receptor A6	Not Reported
1 ENSG00000250251.6	PKD1P6	polycystin 1, transient receptor potential channel interacting pseudogene 6	Not Reported
1 ENSG00000250510.8	GPR162	G protein-coupled receptor 162	Not Reported
1 ENSG00000254838.5	GVINP1	GTPase, very large interferon inducible pseudogene 1	Not Reported
1 ENSG00000254860.6	TMEM9B-AS1	TMEM9B antisense RNA 1	Not Reported
1 ENSG00000255441.1	AC008750.2	novel transcript, antisense to SIGLEC10	Not Reported
1 ENSG00000260828.1	HMGB3P32	high mobility group box 3 pseudogene 32	Not Reported
1 ENSG00000261087.1	ZNNT1	ZNF706 neighboring transcript 1	Not Reported
1 ENSG00000263961.8	RHEX	regulator of hemoglobinization and erythroid cell expansion	Not Reported

1 ENSG00000269403.1	AC008750.8	novel protein	Not Reported
1 ENSG00000272668.2	AL590560.3	novel transcript, antisense to VSIG8	Not Reported
1 ENSG00000272688.1	AP005329.3	novel transcript	Not Reported
1 ENSG00000272908.1	AC006033.2	novel transcript	Not Reported
1 ENSG00000274536.7	AL034397.3	novel transcript, antisense VSIG4	Not Reported
1 ENSG00000276550.4	HERC2P2	hect domain and RLD 2 pseudogene 2	Not Reported
1 ENSG00000278195.2	SSTR3	somatostatin receptor 3	Not Reported
1 ENSG00000278420.1	MIR6819	microRNA 6819	Not Reported
1 ENSG00000280670.3	CCDC163	coiled-coil domain containing 163	Not Reported
1 ENSG00000280832.1	GSEC	G-quadruplex forming sequence containing lncRNA	Not Reported
1 ENSG00000287200.1	AC022506.2	novel transcript, antisense to INHBEand GLI1	Not Reported

ID - DOWN GENES	Symbol	Description	Literature review
1 ENSG00000049323.16	LTBP1	latent transforming growth factor beta binding protein 1	Not Reported
1 ENSG00000071539.14	TRIP13	thyroid hormone receptor interactor 13	Not Reported
1 ENSG00000073282.13	TP63	tumor protein p63	Not Reported
1 ENSG00000076706.17	MCAM	melanoma cell adhesion molecule	Not Reported
1 ENSG00000081041.9	CXCL2	C-X-C motif chemokine ligand 2	Not Reported
1 ENSG00000085840.13	ORC1	origin recognition complex subunit 1	Not Reported
1 ENSG00000088899.15	LZTS3	leucine zipper tumor suppressor family member 3	Not Reported
1 ENSG00000094804.12	CDC6	cell division cycle 6	Not Reported
1 ENSG00000100906.10	NFKBIA	NFKB inhibitor alpha	Not Reported
1 ENSG00000102554.14	KLF5	Kruppel like factor 5	Not Reported
1 ENSG00000102678.7	FGF9	fibroblast growth factor 9	Not Reported
1 ENSG00000104660.19	LEPROTL1	leptin receptor overlapping transcript like 1	Not Reported
1 ENSG00000104671.8	DCTN6	dynactin subunit 6	Not Reported
1 ENSG00000104856.14	RELB	RELB proto-oncogene, NF-kB subunit	Not Reported
1 ENSG00000108342.12	CSF3	colony stimulating factor 3	Not Reported
1 ENSG00000109805.10	NCAPG	non-SMC condensin I complex subunit G	Not Reported
1 ENSG00000110777.12	POU2AF1	POU class 2 homeobox associating factor 1	Not Reported
1 ENSG00000111206.12	FOXM1	forkhead box M1	Not Reported
1 ENSG00000112096.18	SOD2	superoxide dismutase 2	Not Reported
1 ENSG00000115008.5	IL1A	interleukin 1 alpha	Not Reported
1 ENSG00000115009.13	CCL20	C-C motif chemokine ligand 20	Not Reported
1 ENSG00000115884.11	SDC1	syndecan 1	Not Reported
1 ENSG00000116717.13	GADD45A	growth arrest and DNA damage inducible alpha	Not Reported
1 ENSG00000117724.13	CENPF	centromere protein F	Not Reported
1 ENSG00000118503.15	TNFAIP3	TNF alpha induced protein 3	Not Reported
1 ENSG00000118985.16	ELL2	elongation factor for RNA polymerase II 2	Not Reported
1 ENSG00000120875.9	DUSP4	dual specificity phosphatase 4	Not Reported
1 ENSG00000121742.19	GJB6	gap junction protein beta 6	Not Reported
1 ENSG00000123342.16	MMP19	matrix metallopeptidase 19	Not Reported
1 ENSG00000123685.9	BATF3	basic leucine zipper ATF-like transcription factor 3	Not Reported
1 ENSG00000124145.6	SDC4	syndecan 4	Not Reported

1 ENSG00000125319.14	HROB	homologous recombination factor with OB-fold	Not Reported
1 ENSG00000125538.12	IL1B	interleukin 1 beta	Not Reported
1 ENSG00000125657.5	TNFSF9	TNF superfamily member 9	Not Reported
1 ENSG00000125726.11	CD70	CD70 molecule	Not Reported
1 ENSG00000127081.14	ZNF484	zinc finger protein 484	Not Reported
1 ENSG00000130340.16	SNX9	sorting nexin 9	Not Reported
1 ENSG00000132819.17	RBM38	RNA binding motif protein 38	Not Reported
1 ENSG00000134107.5	BHLHE40	basic helix-loop-helix family member e40	Not Reported
1 ENSG00000135047.15	CTSL	cathepsin L	Not Reported
1 ENSG00000135476.11	ESPL1	extra spindle pole bodies like 1, separase	Not Reported
1 ENSG00000136244.12	IL6	interleukin 6	Not Reported
1 ENSG00000136603.14	SKIL	SKI like proto-oncogene	Not Reported
1 ENSG00000136689.18	IL1RN	interleukin 1 receptor antagonist	Philip Kam Weng Kwan, 2020- Active TB vs healthy controls/IGRA negative.<br><br>Lee SW, 2016 - Down LTBI compared to active TB
1 ENSG00000138641.18	HERC3	HECT and RLD domain containing E3 ubiquitin protein ligase 3	Not Reported
1 ENSG00000139289.13	PHLDA1	pleckstrin homology like domain family A member 1	Not Reported
1 ENSG00000139572.4	GPR84	G protein-coupled receptor 84	Not Reported
1 ENSG00000141655.17	TNFRSF11A	TNF receptor superfamily member 11a	Not Reported
1 ENSG00000143603.19	KCNN3	potassium calcium-activated channel subfamily N member 3	Not Reported
1 ENSG00000145386.10	CCNA2	cyclin A2	Not Reported
1 ENSG00000146670.10	CDCA5	cell division cycle associated 5 [	Not Reported
1 ENSG00000150681.10	RGS18	regulator of G protein signaling 18	Not Reported
1 ENSG00000154099.18	DNAAF1	dynein axonemal assembly factor 1	Not Reported
1 ENSG00000154165.5	GPR15	G protein-coupled receptor 15	Not Reported
1 ENSG00000155380.11	SLC16A1	solute carrier family 16 member 1	Not Reported
1 ENSG00000156113.23	KCNMA1	potassium calcium-activated channel subfamily M alpha 1	Tabone, 2021 - Up in clinical TB compared to control
1 ENSG00000157456.8	CCNB2	cyclin B2	Not Reported

1 ENSG00000158406.4	H4C8	H4 clustered histone 8	Not Reported
1 ENSG00000160789.20	LMNA	lamin A/C	Not Reported
1 ENSG00000161888.11	SPC24	SPC24 component of NDC80 kinetochore complex	Not Reported
1 ENSG00000162433.15	AK4	adenylate kinase 4	Not Reported
1 ENSG00000163600.12	ICOS	inducible T cell costimulator	Not Reported
1 ENSG00000163661.4	PTX3	pentraxin 3	Not Reported
1 ENSG00000163734.4	CXCL3	C-X-C motif chemokine ligand 3	Not Reported
1 ENSG00000164236.12	ANKRD33B	ankyrin repeat domain 33B	Not Reported
1 ENSG00000165410.15	CFL2	cofilin 2	Not Reported
1 ENSG00000165474.8	GJB2	gap junction protein beta 2	Not Reported
1 ENSG00000165997.5	ARL5B	ADP ribosylation factor like GTPase 5B	Not Reported
1 ENSG00000166451.13	CENPN	centromere protein N	Not Reported
1 ENSG00000166592.12	RRAD	RRAD, Ras related glycolysis inhibitor and calcium channel regulator	Not Reported
1 ENSG00000167513.9	CDT1	chromatin licensing and DNA replication factor 1	Not Reported
1 ENSG00000170312.16	CDK1	cyclin dependent kinase 1	Not Reported
1 ENSG00000170989.9	S1PR1	sphingosine-1-phosphate receptor 1	Not Reported
1 ENSG00000171848.15	RRM2	ribonucleotide reductase regulatory subunit M2	Not Reported
1 ENSG00000173451.7	THAP2	THAP domain containing 2	Not Reported
1 ENSG00000175746.6	C15orf54	chromosome 15 open reading frame 54 (putative)	Not Reported
1 ENSG00000176845.13	METRNL	meteorin like, glial cell differentiation regulator	Not Reported
1 ENSG00000178803.12	ADORA2A-AS1	ADORA2A antisense RNA 1	Not Reported
1 ENSG00000180535.3	BHLHA15	basic helix-loop-helix family member a15	Not Reported
1 ENSG00000180611.7	MB21D2	Mab-21 domain containing 2	Not Reported
1 ENSG00000182687.4	GALR2	galanin receptor 2	Not Reported
1 ENSG00000183508.5	TENT5C	terminal nucleotidyltransferase 5C	Not Reported
1 ENSG00000184163.3	C1QTNF12	C1q and TNF related 12	Not Reported
1 ENSG00000184588.18	PDE4B	phosphodiesterase 4B	Not Reported
1 ENSG00000185338.5	SOCS1	suppressor of cytokine signaling 1	Not Reported
1 ENSG00000187699.10	C2orf88	chromosome 2 open reading frame 88	Not Reported
1 ENSG00000189057.11	FAM111B	family with sequence similarity 111 member B	Not Reported

1 ENSG00000197122.11	SRC	SRC proto-oncogene, non-receptor tyrosine kinase	Not Reported
1 ENSG00000197147.13	LRRC8B	leucine rich repeat containing 8 VRAC subunit B	Not Reported
1 ENSG00000197632.9	SERPINB2	serpin family B member 2	Not Reported
1 ENSG00000197860.10	SGTB	small glutamine rich tetratricopeptide repeat containing beta	Not Reported
1 ENSG00000198888.2	MT-ND1	mitochondrially encoded NADH:ubiquinone oxidoreductase core subunit 1	Not Reported
1 ENSG00000204186.10	ZDBF2	zinc finger DBF-type containing 2	Not Reported
1 ENSG00000211597.2	IGKJ1	immunoglobulin kappa joining 1	Not Reported
1 ENSG00000211632.4	IGKV3D-11	immunoglobulin kappa variable 3D-11	Not Reported
1 ENSG00000211638.2	IGLV8-61	immunoglobulin lambda variable 8-61	Not Reported
1 ENSG00000211648.2	IGLV1-47	immunoglobulin lambda variable 1-47	Not Reported
1 ENSG00000211665.3	IGLV3-16	immunoglobulin lambda variable 3-16 [	Not Reported
1 ENSG00000211673.2	IGLV3-1	immunoglobulin lambda variable 3-1	Not Reported
1 ENSG00000211676.2	IGLJ2	immunoglobulin lambda joining 2	Not Reported
1 ENSG00000211679.2	IGLC3	immunoglobulin lambda constant 3 (Kern-Oz+ marker)	Not Reported
1 ENSG00000211935.3	IGHV1-3	immunoglobulin heavy variable 1-3	Not Reported
1 ENSG00000213386.3	AC022217.2	family with sequence similarity 58, member A (FAM58A) pseudogene	Not Reported
1 ENSG00000217801.10	AL390719.1	protein tyrosine phosphatase family pseudogene	Not Reported
1 ENSG00000220749.4	RPL21P28	ribosomal protein L21 pseudogene 28	Not Reported
1 ENSG00000222009.8	BTBD19	BTB domain containing 19	Not Reported
1 ENSG00000224041.3	IGKV3D-15	immunoglobulin kappa variable 3D-15 (gene/pseudogene)	Not Reported
1 ENSG00000227165.9	WDR11-AS1	WDR11 antisense RNA 1	Not Reported
1 ENSG00000229344.1	MTCO2P12	MT-CO2 pseudogene 12	Not Reported
1 ENSG00000230002.3	ALMS1-IT1	ALMS1 intronic transcript 1	Not Reported
1 ENSG00000233968.7	AL157895.2	novel transcript, antisense to C10orf112	Not Reported
1 ENSG00000234618.1	RPSAP9	ribosomal protein SA pseudogene 9	Not Reported
1 ENSG00000235065.1	RPL24P2	ribosomal protein L24 pseudogene 2	Not Reported
1 ENSG00000237649.8	KIFC1	kinesin family member C1	Not Reported
1 ENSG00000238039.1	AC244197.2	novel transcript	Not Reported
1 ENSG00000238201.1	AC114752.2	novel transcript	Not Reported
1 ENSG00000239975.2	IGKV1D-33	immunoglobulin kappa variable 1D-33	Not Reported
1 ENSG00000242076.2	IGKV1-33	immunoglobulin kappa variable 1-33	Not Reported
1 ENSG00000253320.7	AZIN1-AS1	AZIN1 antisense RNA 1	Not Reported
1 ENSG00000258572.1	AL133467.1	novel transcript	Not Reported
1 ENSG00000260708.1	AL118516.1	novel transcript, antisense to TBC1D22A	Not Reported
1 ENSG00000267365.1	KCNJ2-AS1	KCNJ2 antisense RNA 1	Not Reported
1 ENSG00000270681.1	AC095055.1	novel transcript, antisense to SH3D19	Not Reported
1 ENSG00000271781.1	AC026740.1	novel transcript, antisense to TPPP	Not Reported

1	ENSG00000273951.1	AL031667.3	novel transcript, sense intronic to CHD6	Not Reported
1	ENSG00000274286.2	ADRA2B	adrenoceptor alpha 2B	Not Reported
1	ENSG00000276070.5	CCL4L2	C-C motif chemokine ligand 4 like 2	Not Reported
1	ENSG00000277075.2	H2AC8	H2A clustered histone 8	Not Reported
1	ENSG00000277632.2	CCL3	C-C motif chemokine ligand 3	Not Reported
1	ENSG00000278356.1	AC005911.1	novel transcript	Not Reported
1	ENSG00000280079.1	AC011447.7	TEC	Not Reported
1	ENSG00000286330.1	AL353660.1	novel transcript	Not Reported

Shared genes

ELTBI vs ATB - DOWN GENES	SYMBOL	Literature review
ENSG00000130812.10	ANGPTL6	Not Reported
ENSG00000131400.8	NAPSA	Not Reported
ENSG00000211898.7	IGHD	Not Reported

ELTBI vs ATB - UP GENES	SYMBOL	Literature review
NSG00000090104.12	RGS1	Not Reported
ENSG00000125538.12	IL1B	Not Reported
ENSG00000136603.14	SKIL	Not Reported
ENSG00000144290.17	SLC4A10	Not Reported
ENSG00000165997.5	ARL5B	Not Reported
ENSG00000173391.9	OLR1	Not Reported
ENSG00000185338.5	SOCS1	Not Reported
ENSG00000196126.11	HLA-DRB1	Not Reported
ENSG00000217801.10	AL390719.1	Not Reported

LLTBI vs ATB - UP GENES	SYMBOL	Literature review
ENSG00000079385.22	CEACAM1	Not Reported

LLTBI vs ATB - DOWN GENES	Not genes found.

Pathway results

upregulated genes

Group	Reactome pathway	Adjusted p-value	Number of upregulated genes
**ATB**	N/A	N/A	N/A
**NI-SI**	Cell Cycle, Mitotic	4.46E-10	12
	Cell Cycle	1.87E-09	12
	M Phase	4.26E-04	7
	G1/S-Specific Transcription	0.004769504	3
	G1/S Transition	0.013668196	4
	Mitotic G1-G1/S phases	0.015873093	4
	Cell Cycle Checkpoints	0.016116383	5
	S Phase	0.019864498	4
	Mitotic Metaphase and Anaphase	0.030274743	4
	Mitotic Prometaphase	0.030274743	4
	Orc1 removal from chromatin	0.030731979	3
	Separation of Sister Chromatids	0.032364541	4
	Mitotic Anaphase	0.032387762	4
	DNA Damage/Telomere Stress Induced Senescence	0.032661066	3
	Polo-like kinase mediated events	0.037632916	2
	Switching of origins to a post-replicative state	0.039193071	3
**LNI**	N/A	N/A	N/A

**LTBI-SI**	G alpha (i) signalling events	1.55E-04	14
	Cytokine Signaling in Immune system	1.82E-04	20
	Chemokine receptors bind chemokines	1.99E-04	7
	Interleukin-10 signaling	2.85E-04	6
	Class A/1 (Rhodopsin-like receptors)	0.002936423	11
	Cytokine-cytokine receptor interaction	0.002995896	10
	Immune System	0.004273297	29
	RUNX2 regulates osteoblast differentiation	0.00445326	4
	Signaling by Interleukins	0.006434591	12
	Chemokine signaling pathway	0.006730335	8
	GPCR ligand binding	0.007242515	12
	Peptide ligand-binding receptors	0.007306545	8
	RUNX2 regulates bone development	0.00892581	4
	Signaling by GPCR	0.016702907	19
	Signal Transduction	0.043985434	31
**LTBI-LI**	Phosphorylation of Emi1	0.001048194	3

downregulated genes

Group	Reactome pathway	Adjusted p-value	Number of downregulated genes
ATB	N/A	N/A	N/A
NI-SI	N/A	N/A	N/A
LNI	Interleukin-10 signaling	1.75E-06	8
	Cell Cycle, Mitotic	1.09E-05	18
	Cytokine Signaling in Immune system	6.64E-05	21
	Cytokine-cytokine receptor interaction	8.31E-05	12
	Cell Cycle	8.40E-05	18
	Signaling by Interleukins	1.11E-04	15
	G1/S-Specific Transcription	3.65E-04	5
	Rheumatoid arthritis	7.30E-04	7
	G alpha (i) signaling events	0.003807673	12
	Cell cycle	0.004142511	7
	Cyclin A/B1/B2 associated events during G2/M transition	0.004431131	4
	Malaria	0.004509032	5
	NOD-like receptor signaling pathway	0.007828307	5
	Condensation of Prometaphase Chromosomes	0.008137635	3
	M Phase	0.008690615	11
	Phosphorylation of proteins involved in the G2/M transition by Cyclin A:Cdc2 complexes	0.019337922	2
	Cell Cycle Checkpoints	0.020199346	9
	Polo-like kinase mediated events	0.020969303	3
	Resolution of Sister Chromatid Cohesion	0.023064917	6
	G1/S Transition	0.02589064	6
	Osteoclast differentiation	0.026057756	6
	Immune System	0.034098438	27
	Mitotic Prometaphase	0.037753482	7
	G2/M DNA replication checkpoint	0.041781373	2
	RAF-independent MAPK1/3 activation	0.042017212	3

	Mitotic G1-G1/S phases	0.042377925	6
LTBI-SI	Neutrophil degranulation	1.24E-05	19
	Innate Immune System	4.67E-04	25
	Phosphorylation of Emi1	0.008179412	3
	Immune System	0.010287755	34
	Class A/1 (Rhodopsin-like receptors)	0.03461297	11
	Chemokine receptors bind chemokines	0.044200545	5
**LTBI-LI**	N/A	N/A	N/A
