# Supplementary material for: Gene expression profiling identifies candidate biomarkers for new latent tuberculosis infections. A cohort study
Source: PLoS One. 2022 Sep 28;17(9):e0274257. doi: 10.1371/journal.pone.0274257 (PMC9518923; doi:10.1371/journal.pone.0274257)
Supplement: S1 File — (PDF) [file pone.0274257.s004.pdf]

Inclusivity in global research

PLOS' policy on inclusivity in global research aims to improve transparency in the reporting of research performed outside of researchers' own country or community and ensures that PLOS publications reporting global research adhere to high standards for research ethics and authorship. Authors of relevant research articles may be asked to complete the questionnaire below, which outlines ethical, cultural, and scientific considerations specific to inclusivity in global research. This questionnaire may be requested when researchers have travelled to a different country to conduct research, if research uses samples collected in another country, research with Indigenous populations or their lands, or if research is on cultural artefacts. Researchers travelling to another country solely to use laboratory equipment will not normally be required to complete the questionnaire. However, the questionnaire can be requested at the journal's discretion for any submission – if you have been requested to complete this questionnaire by the PLOS journal you submitted to, please do so.

Please complete the questionnaire below and include this as a Supporting Information file with your manuscript. Note that if your paper is accepted for publication, this checklist will be published with your article in the supporting information files. Please ensure that you reference the checklist in the main body of your manuscript. We suggest adding a subsection 'Inclusivity in global research' to your Methods section and adding the following sentence: "Additional information regarding the ethical, cultural, and scientific considerations specific to inclusivity in global research is included in the Supporting Information (SX Checklist)"

The questions have been designed to be applicable to a wide range of study types, and there are subsections for both human subjects research and non-human subjects research. If any of the questions are not relevant to your research please mark them as "N/A" as appropriate.

Ethical considerations, permits and authorship

This section is applicable to all research types.

Provide details as to who granted permissions and/or consent for the study to take place in the Methods section of your manuscript. This should include the names of **all** ethics boards, governmental organizations, community leaders or other bodies that provided approval for the study. If individuals provided approval refer to these people by their role or title but do not list their name(s).

Reported on page number: 4

If there were any deviations from the study protocol after approval was obtained please provide details of these changes in the Methods section of your manuscript.

Reported on page number: it does not apply. There were not deviations from the study protocol approved by the ethics boards.

Did this study involve local collaborators that are residents of the country where the research was conducted or members of the community studied? If you do not have any authors from said communities, please provide an explanation for this below.

Mariana Herrera and Zulma Vanessa Rueda are citizens and were residents of Colombia when the research was conducted.
Mariana did her PhD at Universidad de Antioquia, Medellin, Colombia. She also did her PhD intership for two years at the University of Manitoba, where she perform the RNA extraction, sequencing and analysis of this paper.
Zulma Rueda was the principal investigator of this grant and she was a full-time associate professor at Universidad Pontificia Bolivariana, Medellin, Colombia, until January 2021. She also was an adjuct professor (nil salary) at the University of Manitoba between 2015 – 2021. She is currently working at the University of Manitoba, and she continues as adjunct professor (nil salary) with Universidad Pontificia Bolivariana in Colombia.
Juan Pablo Isaza, Lucelly López and Diana Marin are citizens and currently live in Colombia.
Zulma Rueda, Lucelly López, and Diana Marin have been working with people deprived of liberty in Colombia since 2010.

Everyone listed as an author should meet PLOS' criteria for authorship and all individuals who meet these criteria should be included in the author byline, rather than the acknowledgements. Authorship criteria is based on the International Committee of Medical Journal Editors (ICMJE) Uniform Requirements for Manuscripts Submitted to Biomedical Journals - for further information please see here: https://journals.plos.org/plosone/s/authorship.

Human subjects research (e.g. health research, medical research, cross-cultural psychology)

Did you obtain written informed consent from a representative of the local community or region before the research took place? How did you establish who speaks for the community? Details of written informed consent obtained from study participants should be reported separately in the Methods section of your manuscript.

Ethics Committee of the Universidad Pontificia Bolivariana (July 15, 2015), located in Colombia, approved the project. In addition, The Instituto Nacional Penitenciario y Carcelario (INPEC), in English, the Colombian National Penitentiary and Prison Institute, and the director of each prison, approved the project before it started.

The field team (two nurses) visited the prisons from Monday to Friday. They explained the project, the benefits, risk, samples, test, etc., and invited to participate the people deprived of liberty (PDL). We gave the patients the written consent form and it was explained and signed in the presence of two witnesses. These witnesses were PDL, and they signed the written consent form as well. We never took the consent form in the presence of security guards to avoid any type of undue pressure. After this process we used the data collection forms, applied the tuberculin skin test, and took the samples. At all times the PDL were able to ask questions.

The PDL were able to speak at all time to the field team to share their doubts, concerns, or thoughts. The director for each prison, the health workers into the prison responsible for the tuberculosis program, also talk to us any time they consider.

How did members of the local community provide input on the aims of the research investigation, its methodology, and its anticipated outcome(s)?

It is important to mention that we have been working in prisons since 2010, and we provided training about respiratory symptoms and tuberculosis, as well as other kind of training to healtcare workers, prison staff and PDL. These trainings allowed to strengthen the tuberculosis program within prison, implemented community-based TB program, among other activities. PDL that worked within the community-based program worked with our field team to plan the methodology and all activities, and they were with us all the time during the recruitment and follow-ups.

The aims were established by the principal investigator and the researcher team based on previous projects. Once the project was approved, and before the project started, we formally requested the approval by the regional INPEC and the director of each prison. We submitted the full protocol, data collection form and gave a presentation to faciliate questions and answers. In this meeting participated not only the director of each prison, also representatives of PDL. Then, we went to present the project to the healthcare workers and PDL in prisons. The goal of these massive presentations was that people new about us, many knew us from our previous research, but many other do not. We also present the research project and we let them know that it would start in the next months and that they would see us daily when we start the recruitment.

After disseminating the research project and solving questions and answers, we started to invite individual participants. In this individual meeting we explained the project in detailed, answered questions and answers, and took the written consent form.

When engaging with the local community, how did you ensure that the informed consent documents and other materials could be understood by local stakeholders?

In 2012, during our first research project in prisons, PDL wrote a dictionary with all words and slangs that they used inside the prison for us to understand them. PDL affected by TB also gave us diaries that they wrote about their personal experiences while incarcerated and the meaning of that project for them.

We have used these documents to develop and effective communication among the PDL, researchers and local stakeholders.

All the documents, including the informed consents were written in Spanish and using simple words, and the vocabulary used inside the prison. In any case, technical vocabulary was used.

Stakeholders have been involved since 2010 in all our research, we provided progress reports with preliminary and final findings. The information of all our projects was used to improve the TB program in prisons in Antioquia.

Will the findings of the research be made available in an understandable format to stakeholders in the community where the study was conducted (e.g. via a presentation, summary report, copies of publications, etc.)? Please provide details of how this will be achieved.

PDL that we diagnosed with TB was notified immediately and all of them received treatment in less than 24 after the notification. PDL that became new TST converter was notified immediately, we explained again tuberculosis infection and next steps. People were studied to rule out TB.
Our project finished follow-up in 2018. The processing of the samples and analysis was done in 2019 and 2020.
Due to the COVID-19 pandemic, we were not allowed to enter to prison as there were many restrictions related to COVID-19. Therefore, we will do it once the INPEC allows us to enter again to prison.

In addition, all findings are available for the stakeholders in an understandable format. The PI (ZR) shares the information in this website www.tbcolombia.org

Non-human subjects research using specimens/ animals collected as part of the study, or those housed in archival collections. Examples include archaeology, paleontology, botany and zoology.

Did the permission you obtained from a local authority to perform the study include an agreement on access to outputs and benefit sharing? This may include procedures to enable fair distribution of the benefits and resources arising from the research performed. Please include any details of Prior Informed Consent and Benefit Sharing Agreements obtained. These may be required by field-specific regulations, for example the Convention on Biological Diversity (CBD) and the associated Nagoya Protocol.

It does not apply.

If the material used in your study was imported, please A) provide the year it was imported and B) indicate whether permits were obtained to import/export the materials used, C) provide details of any permits obtained. If this information is not available, please indicate this.

It does not apply.

If you used archival specimens, please state how the material used in your study was acquired by the institute it is held in and provide details of any permits obtained for the original excavations/ sample collection. If this information is not available, please indicate this.

It does not apply.

How was the potential cultural significance of the materials collected in your study to local communities considered in your research design? Were Indigenous peoples and/or local researchers and institutions involved with archaeological excavations / collection of specimens? If so, please provide a description of their involvement.

It does not apply.

If your manuscript includes photographs of human remains please indicate whether authors obtained permission from descendants or affiliated cultural communities to do so.

It does not apply.
